# Mediated and moderated associations between cumulative lifetime stressor exposure, emotional dysregulation, impulsivity, and lifetime alcohol use: A cross-sectional scoping study of UK drinkers

**DOI:** 10.1016/j.jpsychires.2023.06.020

**Published:** 2023-06-16

**Authors:** James M. Clay, Kiera A. Baker, Roxana D. Mezabrovschi, Giacomo Berti, Grant S. Shields, George M. Slavich, Lorenzo D. Stafford, Matthew O. Parker

**Affiliations:** a Department of Psychology, University of Portsmouth, UK; b Canadian Institute for Substance Use Research, University of Victoria, Victoria, Canada; c School of Pharmacy and Biomedical Science, University of Portsmouth, Portsmouth, UK; d Department of Psychological Science, University of Arkansas, Fayetteville, USA; e Department of Psychiatry and Biobehavioral Sciences, University of California, Los Angeles, USA; f Surrey Sleep Research Centre, School of Biosciences and Medicine, Faculty of Health and Medical Sciences, University of Surrey, Guildford, UK

**Keywords:** Stress, Alcohol, Impulsivity, Risk-taking, Addiction

## Abstract

Stress, trait impulsivity, and emotional dysregulation are independent predictors of alcohol use and misuse, but little is known about the potential mechanisms that link these risk factors together. To address this issue, we carried out an exploratory cross-sectional study, on UK-based participants. Our preregistered, hypothesised theoretical framework was that emotional dysregulation mediates the association between cumulative lifetime stressor exposure and lifetime alcohol use. We also hypothesised that heightened impulsivity would strengthen these relations. As hypothesised, emotional dysregulation fully mediated the relation between cumulative lifetime stressor exposure and lifetime alcohol use. Several facets of impulsivity moderated these associations. For example, as levels of negative urgency increased, the associations between cumulative lifetime stressor exposure and emotional dysregulation, emotional dysregulation and lifetime alcohol use, and lifetime stress exposure and lifetime alcohol use, via emotional dysregulation, strengthened. These preliminary findings propose a theoretically framed model which integrates several prominent risk-factors for alcohol misuse, extending prior research and generating interesting and novel lines of enquiry for longitudinal and cross-cultural analyses. The findings also highlight the potential clinical utility of screening for lifetime stress exposure while tailoring personalised treatment interventions.

## 1. Introduction

Alcohol misuse (i.e., hazardous drinking) is a global health concern (World Health Organization, 2022). Alcohol use typically begins during adolescence, which can result in altered brain development as this period is critical in structural and functional maturation of the brain, and continues over the life course (for review, see: Silveri, 2012). In some individuals, alcohol misuse can escalate into an addiction (Saunders et al., 2019). A recent meta-analysis of over 1.6 million people suggested that approximately one-in-five patients who enter the UK health system misuse alcohol, and one in ten are dependent (Roberts et al., 2019). Despite this, treatment rates for people suffering alcohol use disorder (AUD) are low (Mekonen et al., 2021) and current interventions are only modestly effective (Ray et al., 2019). Alcohol misuse is a highly complex, multifaceted disorder, with a wide range of risk factors that may differ between individuals. For example, although stress, emotional dysregulation and trait impulsivity are recognised as independent predictors of alcohol use and misuse, almost nothing is currently known about the potential mechanisms and pathways that link these risk-factors together.

Stress is a risk factor both for alcohol misuse (Jose et al., 2000; Ruisoto and Contador, 2019) and emotional dysregulation (Compton et al., 2013; Paulus et al., 2021). Emotional dysregulation is defined as the inability to identify, understand, accept and appropriately react to unwelcome emotional states (Kaufman et al., 2016). Extensive theoretical and empirical work affirms the that link between stress, emotional dysregulation and the risk for alcohol misuse are the result of dysfunction (including both hypo-and hyper-activation) of hypothalamic-pituitary-adrenal (HPA) axis (al’Absi, 2018; Koob and Kreek, 2007; Koob and Schulkin, 2019; Milivojevic and Sinha, 2018). Repetitive activation of the HPA axis, caused by cumulative lifetime stressor exposure, results in neurophysiological changes to areas associated with emotional processing, stress reactivity, and reward regulation (Casement et al., 2015; Kim et al., 2013). Ultimately, these neurophysiological changes can degrade individuals’ ability to regulate their emotions, putting them at increased risk of ‘self-medicating’ (i.e., compensating) through alcohol misuse.

The association between impulsivity and addiction (e.g., AUD) is also well established (Belin et al., 2008; Dalley and Ersche, 2019; Karlsson Linnér et al., 2021; Kreek et al., 2005; Lee et al., 2019; Voon et al., 2020). Impulsivity is defined as a multidimensional personality trait whereby individuals have the propensity to act without forethought to internal or external stimuli with little to no regard for possible negative consequences related to these actions (Strickland and Johnson, 2020). Several clinical diagnoses in the *Diagnostic and Statistical Manual for Mental Disorders, 5th Edition* (American Psychiatric Association, 2013) include impulsivity as a core diagnostic criterion. Research on many of these diagnoses, such as personality disorder (Garofalo et al., 2018), attention deficit hyperactivity disorder (ADHD) (Retz et al., 2012), and AUD (Herman and Duka, 2019), suggests that although related, both emotional dysregulation and impulsivity independently contribute to these conditions.

**Fig. 1.**
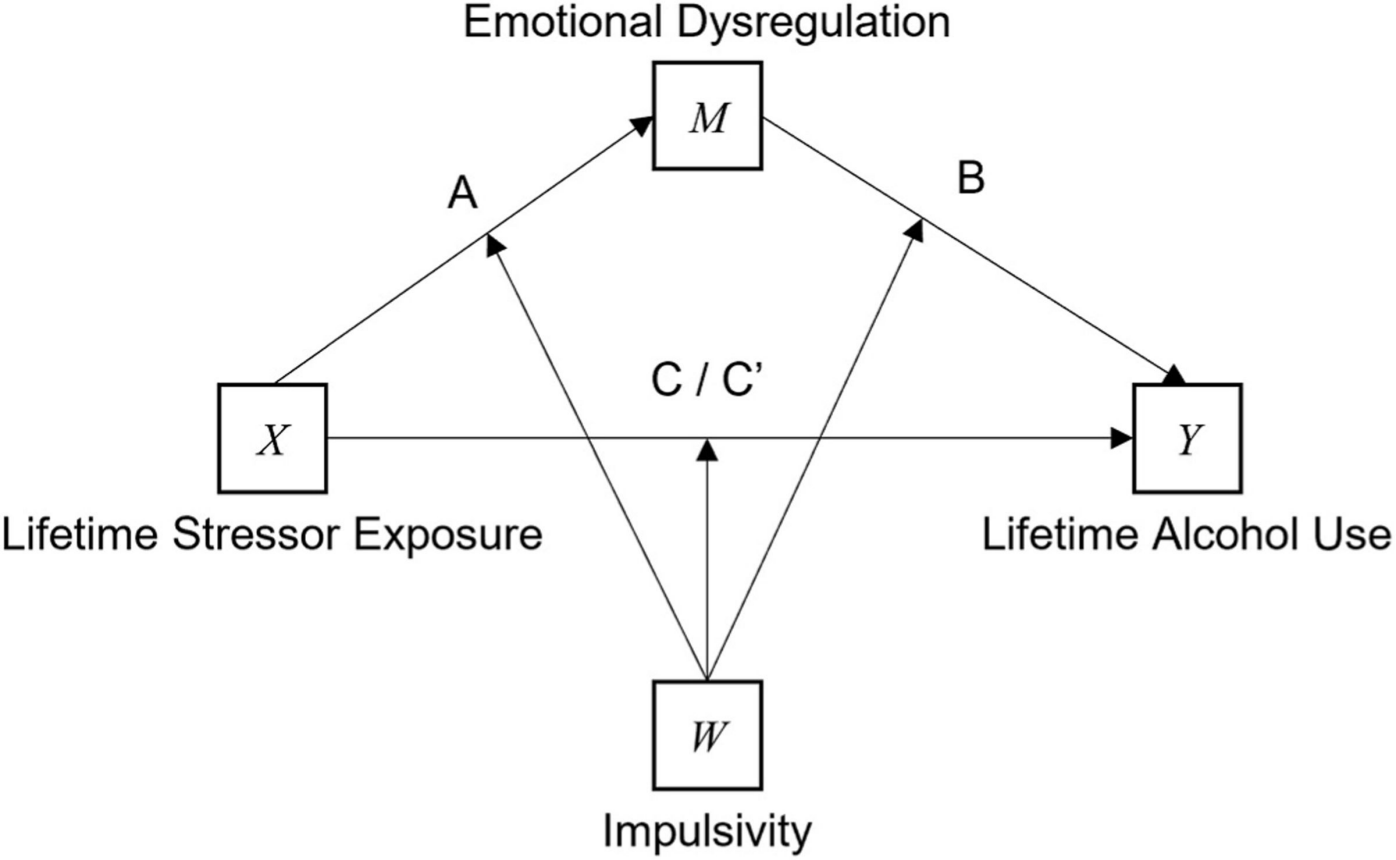
A conceptual diagram illustrating the hypothesised associations between cumulative lifetime stressor exposure (X), emotional dysregulation (M), lifetime alcohol use (Y), and impulsivity (W).

What motivates the present study is prior research from our lab that focussed on how impulsivity may act as a moderator of craving and drinking in times of acute (Clay et al., 2018; Clay and Parker, 2018) and chronic stress (Clay et al., 2021, 2022). Notably, others have focussed on how AUD influences impulsivity via emotional dysregulation (Jakubczyk et al., 2018) or whether the interaction between cumulative lifetime stressor exposure and impulsivity predicts hazardous drinking (Fox et al., 2010). However, no studies have integrated cumulative lifetime stressor exposure, impulsivity and emotional dysregulation into a single model. To address this issue, here we have consolidated the theories described above into a single model (see Fig. 1), which predicts who is most likely to consume increased amounts of alcohol over the life course. Establishing how such clearly defined risk factors for alcohol use fit together into a unified theory will help to better determine personalised interventions which focus on impeding the onset and progression of alcohol misuse and related harms.

To advance the aim of defining a theory-framed, hypothesis-driven model that could predict lifetime alcohol use, we report the results of an initial cross-sectional scoping study, which tested several preregistered hypotheses using conditional process analysis (i.e., moderated mediation analysis). Specifically, our primary hypothesis was that the relation between cumulative lifetime stressor exposure and lifetime alcohol use would be positive and operate via increased emotional dysregulation. We also hypothesised that impulsivity would strengthen these associations.

## 2. Materials and methods

### 2.1. Design

This study used an online cross-sectional design. The independent variable was cumulative lifetime stressor exposure, the mediator variable was emotional dysregulation, the moderator variable was impulsivity, and the dependent variable was lifetime alcohol use.

### 2.2. Transparency and openness

We report how we determined our sample size, all data exclusions, all manipulations, and all measures in the study. A simulation-based sensitivity (Monte Carlo) power analysis (Lakens, 2022) revealed that a minimum of 110 participants were required to achieve sufficient statistical power, (1 – β) = 80%, to test our primary hypothesis (i.e., the mediation effect); see the Supplementary Material for more information. Our final sample size was based on resource constraints (Lakens, 2022). In other words, we collected data from as many participants as we could afford to enable us to address our secondary hypotheses (i.e., the moderation effects). Data and pre-registered hypotheses are posted on the Open Science Framework at https://osf.io/we64c.^1^ Data were analysed using Stata (version 16.1), R (version 4.2.1), and PROCESS for R (version 4.1)

### 2.3. Sample

We selected a sample of 301 adults (152 females, 149 males) from the UK, ranging in age from 18 to 68 years old (*M* = 39.56, *SD* = 12.09), recruited using Prolific Academic (https://www.prolific.co) and reimbursed at a rate of £5/hour. Participants were required to be aged 18 or older; a UK resident; fluent in English; and have a stable internet connection. To bolster the generalisability of the sample, as being an abstainer or heavy drinker is related to higher attrition rates (Torvik et al., 2012), recruitment was stratified by the self-reported UK units of alcohol (10 ml ethanol = 1 unit) consumed per week: 1–4 (25.58%), 5–9 (25.25%), 10–13 (24.58%), and 14+ (24.58%). The study was approved by the University of Portsmouth Science and Health Faculty Ethics Committee (SHFEC, 2021–022A).

### 2.4. Demographic information

Demographic data collected were age, biological sex, relationship status, employment status, student status, highest level of education achieved, past year household income (GBP), and subjective social status using the socioeconomic ladder method (Operario et al., 2004).

### 2.5. Cumulative lifetime stressor exposure

The Stress and Adversity Inventory for Adults (STRAIN) was used to measure cumulative lifetime stressor exposure (Slavich and Shields, 2018). The STRAIN is an online interview that assesses stressful experiences across 55 unique acute and chronic stressors. It uses branching logic to ask follow up questions when a stressor is endorsed (see https://www.strainsetup.com), thus enabling the assessment of both objective (i.e., stressor count) and subjective (i.e., stressor severity) features of major life stressors. The STRAIN has excellent concurrent, discriminant, and incremental validity (Cazassa et al., 2020; Slavich and Shields, 2018; Sturmbauer et al., 2019) and is considered as a ‘gold standard’ assessment (Crosswell and Lockwood, 2020). The stressor severity index captures both the number of stressor exposures that a participant experienced over their entire lifespan and the self-reported severity of each of those stressors.

### 2.6. Emotional dysregulation

We used the 18-item Difficulties in Emotional Regulation Scale Short Form (DERS-SF) to measure emotional dysregulation (Kaufman et al., 2016). The DERS-SF is a standalone scale with six subscales: strategies, non-acceptance, impulse, goals, awareness, and clarity. Participants respond to each item using a five-point Likert scale (1 = *Almost Never*; 5 = *Almost Always*). Therefore, the maximum total score is 90, with higher scores reflecting greater emotional dysregulation. The short form scale retains the excellent psychometric properties of the original scale with half the number of items (Kaufman et al., 2016). The internal consistency was excellent, Cronbach’s α = 0.92.

### 2.7. Alcohol use

#### Alcohol Use Disorders Identification Test (AUDIT).

Participants completed the AUDIT (Babor et al., 1992, 2001) to assess hazardous drinking. It has excellent psychometric properties when used to assess AUDs (Claussen and Aasland, 1993; Fleming et al., 1991). The AUDIT is a ten-item scale, scored on a scale from 0 to 40, where scores between 0 and 7 indicate low-risk drinking, scores between 8 and 15 indicate increasing risk of harm, scores between 16 and 19 higher risk drinking, and a score > 20 suggests alcohol dependence. Internal consistency of the AUDIT was excellent, Cronbach’s α = 0.85.

#### Lifetime Drinking History Questionnaire (LDH-q).

We used the LDH-q to establish participants’ lifetime alcohol use (Friesema et al., 2004). The LDH-q is a validated and reliable tool that captures data about patterns of alcohol use from the onset of regular drinking (defined drinking at least once every three months) across the lifespan (Friesema et al., 2004). Five drinking periods were defined: youth (aged 12–18 years), young adult (aged 19–27 years), adult (aged 28–45 years), middle age (aged 46–60 years), and elderly (aged ≥ 61 years) (Friesema et al., 2004; Lemmens et al., 1997). In each drinking period, participants were asked to record their usual quantity (average units consumed per occasion) and frequency (the number of days per month that the participant drank at this usual level) of drinking. Participants also reported the type of beverage(s) (beer, wine, or sprits) that they consumed, the time of day (morning, afternoon, or evening) that they were drinking, the context (drinking alone or with others) in which they were drinking, and their binge drinking frequency. Using the frequency and quantity data, we calculated the average (expressed as units per week) and total consumption for each phase and across the lifespan.

### 2.8. Impulsivity

As this was a scoping study, impulsivity was assessed using a battery of both self-report and performance-based (i.e., behavioural) measures. We chose a triangulation approach to ensure the broadest translational relevance of our work: impulsivity may be considered as a distinct set of complex constructs, despite commonly being categorised under a single umbrella term (Strickland and Johnson, 2020).

#### Shortened Urgency, Premeditation, Perseverance, Sensation Seeking, Positive Urgency, Impulsive Behaviour Scale (S–UPPSP).

The S–UPPSP was used to assess negative urgency (i.e., the tendency to act rashly under extreme negative emotions), lack of premeditation (i.e., the tendency to act without thinking), lack of perseverance (i.e., the inability to remain focused on a task), sensation seeking (i.e., the tendency to seek out novel and thrilling experiences), and positive urgency (i.e., the tendency to act rashly under extreme positive emotions) (Cyders et al., 2014). The S–UPPSP is a 20-item questionnaire in which participants rate several statements related to their impulsive behaviour on a four-point Likert-type scale (1 = *Agree strongly*; 2 = *Agree some*; 3 = *Disagree some*; 4 = *Disagree strongly*). Each subscale is made up of four items; therefore, the maximum score on each subscale is 16, with higher scores reflecting greater impulsivity. Internal consistency of each subscale ranged from acceptable to good, Cronbach’s α = 0.72 to 0.84.

#### Balloon Analogue Risk Task (BART).

The BART was used to establish risk-taking (Lejuez et al., 2002). The BART, which is a proxy measure of ‘real world’ risk-taking, requires participants to inflate a virtual balloon by pressing the spacebar. Each space bar press earns the participant £0.05 of virtual currency which can be ‘banked’ by pressing the return key. Each balloon has a randomly allocated tolerance and over-inflation will cause the balloon to burst, losing the amount earnt (unbanked) in that trial. An array of 128 numbers were randomly sampled without replacement to set the tolerance of each balloon. As the probability of balloon exploding increases with successive pumps and the task was limited to 30 trials, a selection of trials with a mean burst point of 64 pumps was selected to match that of the original paper (Fernie et al., 2010; Lejuez et al., 2002). The dependent variable for this task is the average number of space-bar presses for *unburst* balloons, reflecting greater risk-taking.

#### Titrating Alternatives Delay Discounting Task (TADD).

Delay discounting (i.e., the reduction in the present value of a future reward as the delay to that reward increases) (Odum, 2011) was assessed using the TADD (Du et al., 2002; Rung et al., 2018). During this task, participants choose either ‘smaller-sooner’ or ‘larger-later’ (e.g., £250 now OR £1, 000 in one year) by pressing the ‘c’ and ‘m’ keys, respectively. In each trial, the smaller-sooner reward was displayed on the left while the larger-later reward was shown on the right. The current delay interval (e.g., “The delay for the options on the right is now 1 WEEK”) for that trial will be displayed at the top of the screen. When the smaller-sooner reward was chosen, the amount of the smaller-sooner reward was reduced by 50% in the subsequent trial. Whereas, if the larger-later reward was chosen, the smaller-sooner reward increased by 50% on the next trial. Overall, this titration procedure was repeated over seven blocks of eight trials, where each block represents a different delay interval (i.e., 1 week, 2 weeks, 1 month, 6 months, 1 year, 5 years, and 25 years). To quantify delay discounting, we calculated both area under the curve (AUC) (Myerson et al., 2001) and *k* (Gray et al., 2016). Unlike *k*, AUC provides a simple atheoretical measure of delay discounting (Bickel and Marsch, 2001; Field et al., 2007), with smaller values (between 0 and 1) reflecting grater delay discounting. Therefore, AUC values were reversed (1 – score), so that greater values represent greater discounting. Prior research has shown the quantification of delay discounting via AUC to be comparable to more conventional curve-fitting techniques (e. g., *k*) (Basile and Toplak, 2015; Odum and Rainaud, 2003). In the present study, a Spearman’s rank correlation indicated a strong relationship between AUC and k, *r*_*s*_ = .87, *p* < .001. Therefore, AUC was used in the analysis.

### 2.9. Procedure

After informed consent was obtained, participants reported their demographic information and then completed the AUDIT. Participants then completed the BART, SST, and TADD in counterbalanced order. Computer tasks were programmed using PsychoPy (Peirce et al., 2019, 2022) and hosted on Pavlovia (https://pavlovia.org/). Next, participants completed the LDH-q, S-UPPSP, and DERS-SF in counterbalanced order using Qualtrics (Provo, Utah). Finally, participants completed the STRAIN, followed by a thank you/debrief message. To ensure data quality, two attention checks (e.g., “It is important that you pay attention to this study. Please select *“Disagree some*”) were embedded in the AUDIT and S-UPPSP. Four participants failed the attention checks and were removed from analyses.

### 2.10. Analysis

Descriptive statistics (means, standard deviations, and the proportion of missing data) were calculated and bivariate associations were explored for key study variables. The proportion of missing data by variable is shown in Table S2. Due to the small proportion of missing data, deletion methods are unlikely to bias the results (Schafer, 1999).

Our primary hypothesis (mediation) was tested using PROCESS model 4^2^. Our secondary hypotheses (moderation) were tested using PROCESS model 59. Bias-corrected bootstrapped (*n* = 10,000) 95% confidence intervals (CIs) were used to test for statistical significance in PROCESS models. Pairwise deletion and listwise deletion was used for correlations and regressions (i.e., PROCESS models), respectively.

Preregistered covariates included: age (Leigh and Stacy, 2004), sex (White et al., 2015), and socioeconomic status (SES) (Probst et al., 2020). In our preregistration, we expected that variables related to SES would load together during factor analysis, enabling us to create an index of SES. However, this was not observed (see Supplementary Material). Instead, we recoded education (GCSE & below, A-levels & equivalent, and Undergraduate & higher), employment (unemployed, student, employed), household income (low < £54,406^3^, medium = £54, 406, high > £54,406), and subjective social status (low < 5, medium = 5, high > 5) into larger groups, to conserve statistical power, and included them in our models as separate variables along with age and sex. Similarly, our impulsivity variables did not load together in a factor analysis (see Supplementary Material); therefore, the models were separated by construct to conserve statistical power and to avoid erroneously conditioning our estimates (Clay et al., 2022; McMullin et al., 2020).

Interactions were probed using the Johnson-Neyman technique (Johnson and Neyman, 1936). Prior to analysis, both univariate and multivariate outliers were screened following Tabachnick and Fidell (2014). Univariate outliers were assessed using *z*-scores, where a *z*-score >3.29 and < −3.29 (*p* < .001, two-tailed test) was considered a univariate outlier (one participant was excluded). The assessment of multivariate outliers was based on a Mahalanobis distance that is significant at the *p* < .001 level, assuming that the test statistic follows a chi-square distribution (Verardi and Dehon, 2010). Results were considered significant when *p* < .05 or when the 95% CI did not contain zero.

## 3. Results

Table 1 presents the sociodemographic characteristics of the sample. Table 2 displays the descriptive statistics (means and standard deviations) for the main study variables in terms of cumulative lifetime stress, emotional dysregulation, alcohol use behaviour, and impulsivity. Further descriptive statistics for alcohol use behaviour variables can be seen in Fig. S3.

### 3.1. Bivariate analysis

As shown in Table S6, AUDIT and lifetime alcohol use were intercorrelated (*r*_s_ = .69, *p* < .001), and were also positively correlated with emotional dysregulation (DERS-SF; *r*_s_ = .24 to 0.41, all *p*_*s*_ < .001), cumulative lifetime stress (STRAIN stressor severity; *r*_s_ = .26 to 0.34, all *p*_s_ < .001), and all measures of self-report impulsivity (S-UPPSP; *r*_s_ = .15 to 0.38, all *p*_s_ < .011), except sensation seeking and perseverance, which were not correlated with lifetime alcohol use (*p*_s_ > .05). There was also a significant positive correlation between delay discounting (1 – AUC) and lifetime alcohol use (*r*_*s*_ = .13, *p* = .025). All measures of *self-reported* impulsivity were intercorrelated (*r*_s_ = .13 to 0.68, all *p*_s_ < .05), except for the relations between premeditation and sensation seeking (*p* = .303) and perseverance and positive urgency (*p* = .134). Surprisingly, a negative correlation between delay discounting and risk-taking (BART) was observed (*r*_s_ = −.12, *p* = .0375).

### 3.2. Emotional dysregulation mediates the relationship between cumulative lifetime stressor exposure and lifetime alcohol use

The results of the mediation analysis are summarised in Table 3. After adjusting for covariates, cumulative lifetime stressor exposure positively predicted emotional dysregulation (*B* = 0.15, β = 0.34, 95% CI = 32.88 to 56.09) and emotional dysregulation positively predicted lifetime alcohol use (*B* = 0.47, β = 0.19, 95% CI = 0.10 to 0.85). Significant indirect (*B* = 0.07, β = 0.06, 95% CI = 0.01 to 0.14) and total (*B* = 0.20, β = 0.18, 95% CI = 0.06 to 0.33) effects were observed, while the direct effect was not significant (*B* = 0.13, β = 0.12, 95% CI = −0.02 to 0.12). Taken together, these results suggest full statistical mediation of the association between cumulative lifetime stress exposure and lifetime alcohol use through cumulative lifetime stress exposure.

### 3.3. Negative urgency is a critical moderator of the cumulative lifetime stressor exposure, emotional dysregulation, lifetime alcohol use pathway

Tables (S7-S13) summarising the output for the conditional process analyses are reported in the Supplementary Material. Moderation analysis suggested that negative urgency modified the association between cumulative lifetime stressor exposure and emotional dysregulation (*B* = 0.02, 95% CI = 0.01 to 0.03) and the association between emotional dysregulation and lifetime alcohol use (*B* = 0.13, 95% CI = 0.01 to 0.26). Lack of perseverance also modified the relation between emotional dysregulation and alcohol use (*B* = 0.21, 95% CI = 0.02 to 0.37), whereas positive urgency modified the association between cumulative lifetime stressor exposure and alcohol use (*B* = −0.05, 95% CI = −0.10 to −0.001).

**Fig. 2.**
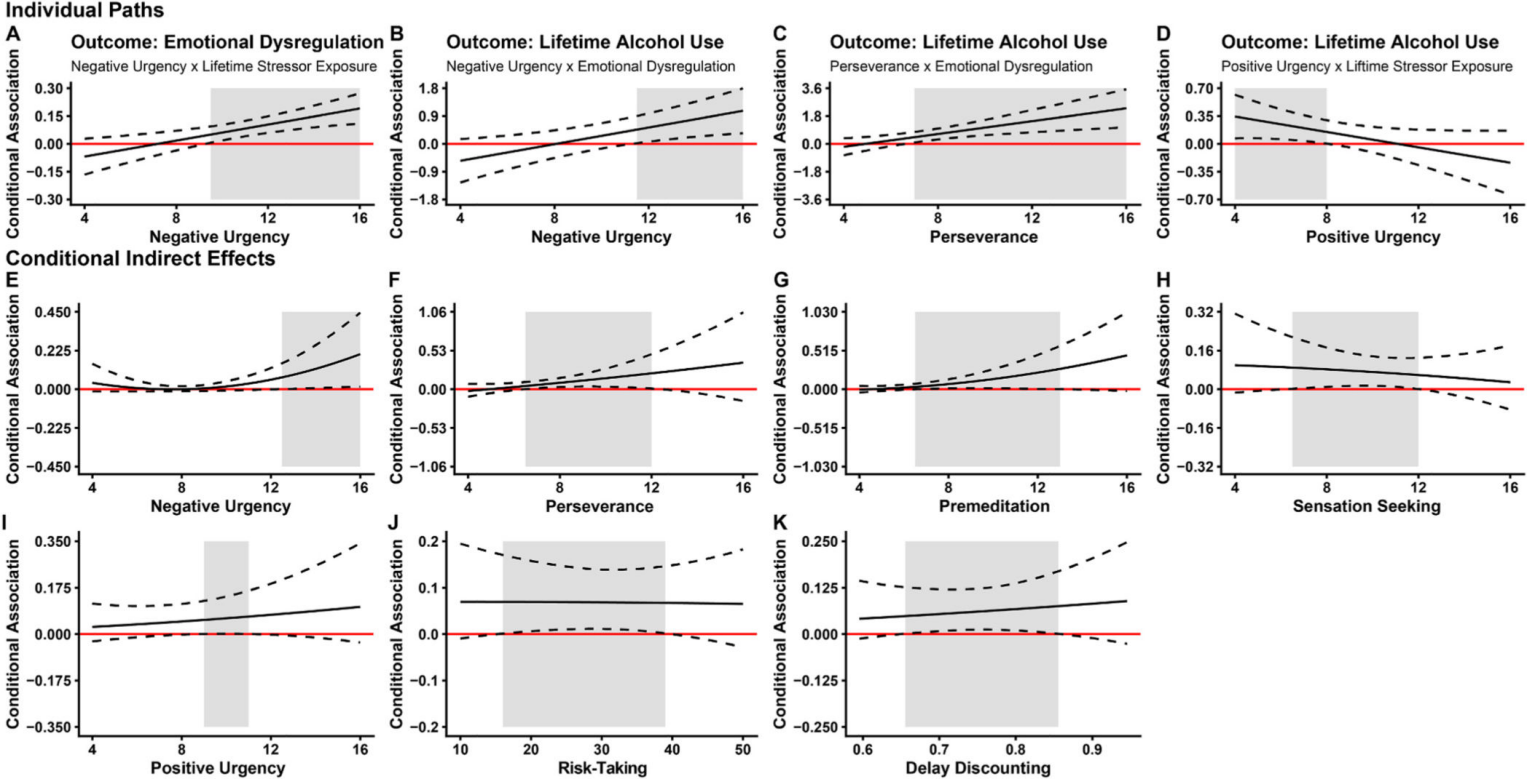
Johnson-Neyman plots illustrating the moderating role of impulsivity measurements in the mediated relation between cumulative lifetime stressor exposure, emotional dysregulation, and lifetime alcohol use. *Note.* The shaded area represents the region of significance (*p* < .05). Emotional dysregulation was measured using the Difficulties in Emotional Regulation Scale Short Form. Cumulative lifetime stressor exposure was assessed using the Stress and Adversity Inventory for Adults. Lifetime alcohol use was measured using the Lifetime Drinking History Questionnaire. Negative and positive urgency, (lack of) perseverance, (lack of) premeditation, and sensation seeking were assessed using the Shortened UPPS–P Impulsive Behaviour Scale. Risk-taking was measured using average number of space bar presses for unburst balloons during the Balloon Analogue Risk Task. Delay discounting was calculated as 1 minus the area under the curve score (so that greater scores reflect greater delay discounting) on the Titrating Alternatives Delay Discounting Task.

Johnson-Neyman plots (see Fig. 2) revealed that associations between cumulative lifetime stressor exposure and emotional dysregulation, emotional dysregulation and alcohol use, and the indirect association (i.e., the relation between cumulative lifetime stressor exposure and lifetime alcohol use, through emotional dysregulation) were strengthened as negatively urgency increased from 9.5, 11.5 and 12.5, respectively. A similar pattern was observed for lack of perseverance (values ≥ 7) and the association between emotional dysregulation and alcohol use. However, the opposite was observed for positive urgency and the association between cumulative lifetime stressor exposure and alcohol use, where the relationship was weakened as positive urgency increased (values ≤ 8 were significant). In terms of other modified indirect effects (Fig. 2, panels F–K), middling values tended to be significant. However, the slopes, as values of impulsivity increased, were relatively less steep. Therefore, these findings suggest that moderators in Fig. 2 F–K were relatively less effective in predicting lifetime alcohol use.

## 4. Discussion

We tested a theoretically-driven model of risk factors for lifetime alcohol use in this study. Specifically, we aimed to determine: (a) if emotional dysregulation mediates the relation between cumulative lifetime stressor exposure and lifetime alcohol use; and (b) whether these associations were strengthened by greater impulsivity, operationally defined using both self-report and behavioural methods. Consistent with our preregistered hypotheses, we found statistical evidence that emotional dysregulation fully mediated the association between cumulative lifetime stressor exposure and lifetime alcohol use, demonstrated by a significant indirect (ab) effect and non-significant direct effect (c). We also found that urgency (both negative and positive) and perseverance are crucial moderators of these associations. Contrary to our hypothesis, self-report premeditation and sensation seeking, and our behavioural measures of impulsivity, were less useful regarding the prediction of lifetime alcohol use.

The individual contributions of stressor exposure, emotional dysregulation and impulsivity to increased alcohol use are well established (Blaine and Sinha, 2017; Carbia et al., 2021). Consistent with this research, we found that greater self-reported impulsivity was independently related to increased AUDIT score (except sensation seeking) and lifetime alcohol use (except sensation seeking and perseverance). Similarly, we found that that behavioural delay discounting (1 – AUC) was associated with increased lifetime alcohol use. In contrast, behavioural risk-taking (BART) was not correlated with alcohol use behaviour.

There is clear evidence that stressor exposure causes emotional dysregulation (Sapolsky, 2007); that emotional dysregulation is greater in alcohol-dependent individuals (Sinha, 2012); and that impulsivity is a personality trait (Cyders et al., 2014), which is likely to manifest during development (Niv et al., 2012). To our knowledge, this is the first study to demonstrate that emotional dysregulation fully mediates the relation between cumulative lifetime stressor exposure and lifetime alcohol use. Moreover, it is the first to determine the different facets of impulsivity that moderate this mediated association. Collectively, this provides evidence that our variables of interest are temporally spaced, giving confidence that longitudinal follow-up studies would be likely to show similar results (Hayes, 2022). We argue that further testing of our model would lead to fruitful theoretical and, potentially, therapeutic advances. For instance, interventions which aim to improve emotional regulation may be beneficial in prevention and treatment efforts.

No studies have investigated mediated associations between stress, impulsivity, emotional dysregulation and alcohol use behaviour. However, Hamilton et al. (2013) tested multiple stress → impulsivity → hazardous drinking models, iterating over several stressor types, and found that self-report impulsivity *partially* mediated the relation between cumulative lifetime stress and alcohol use behaviour. Similarly, Kim et al. (2013) suggest that ‘reflection impulsivity’, ‘response impulsivity’, and ‘aggression’ *partially* mediate the association between early life stress and alcohol dependence. Here, we specified a cumulative lifetime stressor exposure → emotional dysregulation → lifetime alcohol use mode, providing evidence for *full* mediation. Finally, as Jakubczyk et al. (2018) found evidence that emotional dysregulation *partially* mediated the relation between AUD symptomology and increased impulsivity, it is probable that stressor exposure, emotion dysregulation and impulsivity are both risk-factors for, and consequences of, alcohol misuse. However, due to the cross-sectional design used in these studies, and here, it is impossible to determine directionality or causality. This should be an area of future research focus.

The interactive effects of impulsivity and acute (Clay et al., 2018; Clay and Parker, 2018), chronic (Clay et al., 2021, 2022), and cumulative (Fox et al., 2010) stress on alcohol use behaviour have been previously reported but not in the context of mediation. We found that the positive associations between cumulative lifetime stressor exposure and emotional dysregulation; emotional dysregulation and lifetime alcohol use; and cumulative lifetime stressor exposure and lifetime alcohol use, through emotional dysregulation were strengthened as values of negative urgency increased. Similarly, the association between emotional dysregulation and lifetime alcohol use was strengthened as (lack of) perseverance increased. Meanwhile – and as expected, given the pattern of negative urgency findings – the relation between cumulative lifetime stressor exposure and lifetime alcohol use became weaker as levels of positive urgency increased. Finally, all other measures of inhibitory impulsivity, except sensation seeking and risk-taking (BART), strengthened the indirect effect. However, the moderation slopes were less steep (vs. negative urgency), and middling values tended to be significant. Therefore, we conclude that these measures are perhaps a less useful target for future research focus compared to negative urgency.

The biological mechanisms underlying these patterns remain unclear. However, several stress-related changes in biology could partly explain our findings. For instance, as stress exposure(s) cumulates over an individual’s life, HPA axis sympathetic-adrenal-medullary axis, and systemic inflammatory activity is upregulated (Graham et al., 2006; Lupien et al., 2009), leading to increased allostatic load (i.e., biological ‘wear and tear’) (McEwen, 1998) and the associated risk for diseases, disorders and death (Lupien et al., 2009). People who begin to misuse alcohol may do so in an attempt to ‘self-medicate’. However, as the hedonic effects wear off, their allostatic load is increased further by the distress of withdrawal, and overtime, after repeated binges, a change in their allostatic set-point leaves them vulnerable to alcohol misuse and related harm (Koob, 2001).

Furthermore, impulsivity and emotional dysregulation are thought to be partly heritable (Niv et al., 2012; Rappaport et al., 2020) and functional magnetic resonance imaging (fMRI) and event related potential (ERP) studies show that both emotional regulation and impulsivity share overlapping networks, situated predominantly in the prefrontal cortex (Brown et al., 2012; Messerotti Benvenuti et al., 2015). Therefore, it may be that those who are high in trait-impulsivity (particularly urgency) expend a great deal of cognitive resources on emotional processing, leaving limited resources for decision making (Jakubczyk et al., 2018; Seo et al., 2016). Ultimately, resulting in maladaptive decisions, such as alcohol misuse.

An alternative explanation is that, in line with the stress generation hypothesis (Hammen, 2006), those high in negative urgency tend to experience a greater number of negative dependent events (Liu and Kleiman, 2012). Similarly, negative urgency has been shown to moderate acute stress reactivity (Owens et al., 2018). Therefore, those high in negative urgency may exacerbate current, or generate new, stressful life events. Put differently, life may be more stressful for those high in negative urgency. This may help to explain why meta-analysis results show that negative urgency is one of the strongest impulsivity-related correlates of alcohol-related problems and dependence (Coskunpinar et al., 2013) and, in the present study, the model containing negative urgency explained 51% of the variance in emotional dysregulation and 14% of the variance in lifetime alcohol use. Therefore, interventions focussed on reducing negative urgency may prove useful.

## 5. Limitations

We acknowledge several limitations. First, although the cross-sectional mediation analysis provides initial support for our hypothesis, without complementary longitudinal analyses we cannot make firm conclusions regarding causality or temporal onset (Hayes and Rockwood, 2020). Second, due to technical limitations (i.e., having to pass participants between software systems), our measures were not fully counterbalanced (i.e., the STRAIN was always completed last). This may have caused uncontrolled order effects. It should be noted, however, that measures were counterbalanced within blocks and the most cognitively demanding tasks (i.e., the behavioural computer tasks) were presented at the beginning of the study. Third, our stop-signal task data (see Supplementary Material) was unreliable and the psychometric properties of the BART have been questioned in prior research (Steiner and Frey, 2021). Fourth, self-report measures are prone to measurement error owing to reliance on participants’ accurate memory and/or response biases and demand characteristics. For example, individuals typically under-estimate their alcohol consumption during questionnaires (Northcote and Livingston, 2011) and self-report impulsivity measures may reflect self-identified behaviours rather than the construct that is intended to be assessed (Lane et al., 2003). Therefore, future research should focus on creating behavioural measurement of UPPS-P constructs, which would also enable subsequent translational (i.e., animal) research. Finally, there are other potential confounding factors that were not accounted for here as these data were not available. For instance, psychopharmacological drugs (e.g., antidepressants and stimulants) can alter mood (Jakubovski et al., 2019) and reduce impulsivity (Grant and Chamberlain, 2015). Acquiring such data would have come at the cost of reduced statistical power and increased participant burden. Thus, it was not feasible within the scope of this work.

## 6. Conclusion

In conclusion, the present cross-sectional scoping study extends prior research by testing a theoretically driven model of alcohol use. We found evidence to suggest that individuals who have higher cumulative lifetime stressor exposure tend to have higher alcohol use due to also having higher levels of emotional dysregulation. Furthermore, these relations were stronger in those with high negative urgency. These findings have important implications for both researchers and clinicians. These data highlight the potential clinical utility for lifetime stress exposure screening and identify potential targets for personalised treatment interventions. For example, treatment interventions which improve emotional regulation ability and/or reduce negative urgency may prove beneficial for decreasing alcohol use and misuse.

## Supplementary Material

Supplementary Material

## Figures and Tables

**Table 1:** Sociodemographic characteristics of the sample.

Variable	Total (*SD*)	Female (*SD*)	Male (*SD*)

*N*	296	150	146
Age	39.60 (12.11)	39.44 (12.08)	39.76 (12.18)
Average units per week			
1–4	25.68%	26.00%	25.34%
5–9	25.00%	24.00%	26.03%
10–13	24.32%	24.67%	23.97%
14+	25.00%	25.33%	24.66%
Relationship Status			
Divorced	3.38%	6.00%	0.68%
Engaged	4.73%	6.00%	3.42%
Civil partnership	1.01%	2.00%	0.00%
In a relationship	28.72%	31.33%	26.03%
Married	34.46%	29.33%	39.73%
Never married	0.68%	0.00%	1.37%
Separated	1.69%	2.00%	1.37%
Single	20.95%	19.33%	22.60%
Widowed	1.01%	1.33%	0.68%
Employment			
Unemployed	4.73%	6.00%	3.42%
Student	16.55%	16.00%	17.12%
Employed	75.34%	76.00%	74.66%
Education			
No qualifications	0.34%	0.67%	0.00%
GCSE	6.76%	6.00%	7.53%
A-levels	13.85%	15.33%	12.33%
Technical college	14.19%	12.00%	16.44%
Undergraduate degree	37.84%	37.33%	38.36%
Graduate degree	20.95%	21.33%	20.55%
Doctorate degree	4.73%	5.33%	4.11%
Household Income			
< £10,000	3.38%	4.00%	2.74%
£10,000 - £15,999	3.72%	4.67%	2.74%
£16,000 - £19,999	6.76%	8.00%	5.48%
£20,000 - £29,999	16.22%	17.33%	15.07%
£30,000 - £39,999	18.24%	20.67%	15.75%
£40,000 - £49,999	16.55%	15.33%	17.81%
£50,000 - £59,999	10.14%	7.33%	13.01%
£60,000 - £69,999	8.45%	5.33%	11.64%
£70,000 - £79,999	4.73%	3.33%	6.16%
£80,000 - £89,999	3.38%	2.67%	4.11%
£90,000 - £99,999	3.04%	4.67%	1.37%
£100,000 - £149,999	4.39%	5.33%	3.42%
> £150,000	1.01%	1.33%	0.68%
Subjective Social Status (Socioeconomic Ladder)			
1	0.34%	0.00%	0.68%
2	1.01%	0.67%	1.37%
3	5.74%	6.00%	5.48%
4	16.22%	18.00%	14.38%
5	20.27%	22.00%	18.49%
6	25.68%	24.67%	26.71%
7	22.97%	22.67%	23.29%
8	6.42%	6.00%	6.85%
9	1.35%	0.00%	2.74%
10	0.00%	0.00%	0.00%

*Note.* Data are presented as mean (SD) for continuous measures and % for categorical measures.

**Table 2:** Descriptive statistics (M and SD) for main study variables.

Variable	Total (*SD*)	Female (*SD*)	Male (*SD*)

Lifetime Stressor Count (STRAIN)	18.24 (12.24)	20.77 (12.92)	15.64 (10.94)
Lifetime Stressor Severity (STRAIN)	45.10 (30.94)	52.58 (33.22)	37.41 (26.38)
DERS-SF Total	42.80 (13.15)	44.12 (13.20)	41.45 (13.01)
AUDIT	11.87 (6.89)	12.39 (7.24)	11.34 (6.50)
Weekly Consumption (UK Units)	31.56 (33.69)	29.61 (33.84)	33.57 (33.52)
SUPPS-P Negative Urgency	9.50 (3.08)	10.01 (3.03)	8.97 (3.05)
SUPPS-P Premeditation	7.37 (2.06)	7.49 (2.11)	7.25 (2.02)
SUPPS-P Perseverance	7.06 (2.19)	7.44 (2.18)	6.66 (2.13)
SUPPS-P Sensation Seeking	9.73 (2.87)	9.15 (2.93)	10.32 (2.68)
SUPPS-P Positive Urgency	7.54 (2.74)	7.58 (2.70)	7.51 (2.78)
BART	29.12 (11.90)	29.34 (11.96)	28.91 (11.87)
1 - AUC	0.77 (0.12)	0.78 (0.12)	0.77 (0.12)

Note. STRAIN = Stress and Adversity Inventory for Adults; DERS-SF = Difficulties in Emotional Regulation Scale Short Form; AUDIT =Alcohol Use Disorders Identification Test; 1 unit = 8g pure ethanol; SUPPS-P = Shortened Urgency, Premeditation (lack of), Perseverance (lack of), Sensation Seeking, Positive Urgency, Impulsive Behaviour Scale; BART = average number of space bar presses for unburst balloons during the Balloon Analogue Risk Task; 1 – AUC = 1 minus the area under the curve score (so that greater scores reflect greater delay discounting) for the Titrating Alternatives Delay Discounting Task.

**Table 3:** Summary of the mediation analysis examining whether emotional dysregulation mediates the effect between cumulative lifetime stress and lifetime alcohol use (N = 279).

Antecedent		Consequent										
		
		*M* (DERS-SF)						*Y* (Alcohol Use)				
				
		B	β	SE	LL	UL		B	β	SE	LL	UL

Constant	*i*_*M*_	**44.02**		**5.91**	**32.88**	**56.09**	*i*_*ϒ*_	32.60		21.44	−6.34	78.27
*X* (STRAIN)	*a*	**0.15**	**0.34**	**0.03**	**0.10**	**0.20**	*c’*	0.13	0.12	0.07	−0.005	0.26
*M* (DERS-SF)		–	–	–	–	–	*b*	**0.47**	**0.19**	**0.19**	**0.10**	**0.85**
Age		**−0.34**	**−0.31**	**0.06**	**−0.45**	**−0.22**		−0.06	−0.02	0.17	−0.39	0.27
Sex = Male		−0.56	**−**0.02	1.47	−3.45	2.32		6.74	0.10	3.91	−1.09	14.34
Education												
GCSE & below		Ref.						Ref.				
A-levels & equivalent		4.75	0.16	3.32	−1.86	11.28		**−17.89**	**−0.24**	**9.36**	**−37.40**	**−0.55**
Undergraduate & higher		2.75	0.10	3.08	−3.44	8.75		**−19.55**	**−0.28**	**9.07**	**−38.76**	**−3.00**
Employment												
Unemployed		Ref.						Ref.				
Student		5.42	0.16	3.77	−2.20	12.66		−6.21	−0.07	17.19	−43.48	21.81
Employed		4.89	0.15	2.96	−1.24	10.37		−12.38	−0.15	15.60	−47.21	11.06
Household Income												
Low		Ref.						Ref.				
Medium		−1.33	−0.03	2.21	−5.47	3.18		−1.93	−0.02	5.01	−11.29	8.37
High		−1.56	−0.05	1.79	−4.98	2.06		0.02	0.00	4.95	−9.23	10.12
Subjective Social Status												
Low		Ref.						Ref.				
Medium		−0.91	−0.03	2.18	−5.23	3.25		2.56	0.03	6.73	−9.77	16.66
High		−2.08	−0.08	1.91	−5.82	1.69		−0.01	0.00	4.73	−9.12	9.29
		R^2^ = 0.22						R^2^ = 0.10				
		*F*(11, 267) = 7.13, *p* < .001						*F*(12, 266) = 2.64, *p* = .002				

*Note.* Models were adjusted for age, sex, highest level of education achieved, employment status, and household income. LL and UL represent the lower and upper limit of the bootstrapped 95% CI (10,000 bootstraps), respectively. STRAIN = Stress and Adversity Inventory for Adults Stressor Severity Index; DERS-SF = Difficulties in Emotional Regulation Scale Short Form; 1 unit = 8g pure ethanol. Significant effects (*p* < .05) are in boldface.

## Footnotes

1 We originally planned to include the Stop Signal Task as an additional behavioural measure of impulsivity (see our preregistration). However, the task parameters indicated that this data was unreliable according to Verbruggen et al. (2019). Therefore, the task was omitted from analyses. Towards transparency and openness, we report the task procedure, task parameters, and rationale for omitting this measure from the analyses in the Supplementary Material.

2 The original preregistration stated that structural equation modelling (SEM) would be used to test our hypotheses. However, as conditional process analyses (i.e., PROCESS models) are easier to implement and the results are largely identical, thus the choice between the two are inconsequential (Hayes et al., 2017),we chose to use PROCESS to analyse our data.

3 The median household income in the UK for the financial year ending in 2021 was £54,406 (Office for National Statistics, 2022).
