# Supplementary material for: Mediated and moderated associations between cumulative lifetime stressor exposure, emotional dysregulation, impulsivity, and lifetime alcohol use: A cross-sectional scoping study of UK drinkers
Source: J Psychiatr Res. Author manuscript; Available in PMC 2023 Dec 28. (PMC10754206; doi:10.1016/j.jpsychires.2023.06.020)
Supplement: Supplementary Material [file NIHMS1911088-supplement-Supplementary_Material.docx]

Supplement to:

Mediated and Moderated Associations Between Cumulative Lifetime Stressor Exposure, Emotional Dysregulation, Impulsivity, and Lifetime Alcohol Use: A Cross-Sectional Scoping Study of UK Drinkers

## **Power analysis**

The following procedure is partially described in Schoemann et al. (2017) and Lakens (2022). We implemented a simulation-based sensitivity (Monte Carlo) power analysis using an online Shiny App (<https://schoemanna.shinyapps.io/mc_power_med/>). The effect sizes entered into the power analysis were informed by taking the mean of correlation coefficients reported in previous literature. We assumed correlations of .283 for the relationship between *X* and *M* (Abravanel and Sinha, 2015; Burns et al., 2010); 449 for the relationship between *M* and *Y* (Aurora and Klanecky, 2016; Dragan, 2015; Khosravani et al., 2017; Mandavia et al., 2016; Petit et al., 2015); and .224 between *X* and *Y* (Dawson et al., 2005; Fox et al., 2010; Mandavia et al., 2016). 110 participants were required to achieve sufficient statistical power, (1 – β) = 80%, to test our primary hypothesis (mediation effect). As we also planned to address our secondary hypotheses (moderation effects), we collected data from as many participants as our financial resources would allow (Lakens, 2022).

## Stop Signal Task (SST)

Participants completed the SST to assess response inhibition (i.e., the ability to withhold inappropriate action) (Lappin and Eriksen, 1966; Logan and Cowan, 1984; Verbruggen et al., 2019). Participants were instructed to *“Respond to an arrow, which is surrounded by a white circle, pointing either left or right, as fast as you can…However, if the arrow is surrounded by a RED CIRCLE, you should NOT RESPOND”.* Each trial began with a white fixation cross in the centre of the screen which remained for 500ms followed by a 500ms inter-stimulus interval. Next, an arrow pointing either left or right was presented for 1000ms in the middle of the frame. In ‘go’ trials, participants were required to respond by indicating if an arrow surrounded by a white circle (go-signal) was pointing left (with the ‘c’ button on a keyboard) or right (with the ‘m’ button). In ‘stop signal’ trials, the arrow was surrounded by a red circle (stop-signal) after a brief delay, and participants were instructed to withhold their response.

During the first stop trial, the stop-signal occurred 50ms after the presentation of the go-signal and remained for the remaining duration of the trial (950ms). In subsequent trials, the latency of the onset and the corresponding duration of the stop-signal varied (by 100ms) based on how the participant performed during the previous stop-signal trial; where the maximum and minimum values for the latency of the onset of a stop-signal was 350ms and 50ms, respectively. Therefore, there were four possible values: 50ms, 150ms, 250ms, 350ms. Finally, participants were presented with visual feedback indicating how they performed on the previous trial. After a correct response, the feedback message said *“Correct”*, following an incorrect response to a go-signal (i.e., not responding), the feedback message said *“You should have pressed!”*, and after an incorrect response to a stop-signal, the feedback message said *“You should NOT have pressed!”*. Overall, participants completed an initial training block of 16 trials (12 go trials and 4 stop trials) followed by an experimental block of 200 trials (150 go trials and 50 stop trials). Each block was fully randomised. The main dependent variable for the SST is the stop signal reaction time (SSRT).

We report several task-related descriptive statistics in Table S1, following *“A consensus guide to capturing the ability to inhibit actions and impulsive behaviors in the stop-signal task”* (Verbruggen et al., 2019). P(Response|Signal) should ≈ 0.50 (Band et al., 2003) and, at the very least, individual SSRTs should *not* be estimated when P(Response|Signal) is lower than 0.25 or higher than 0.75 (Congdon et al., 2012). Applying this rule in the present study resulted in 58.11% of the data being excluded, suggesting that the SST data were unreliable for the majority of the participants. We did not include this measure in our analyses due to the questionable reliability of the data in the present study and the reduction in sample size and statistical power following the exclusion of the unreliable data.

**Table S1**

*Stop Signal Task Descriptive Statistics*

| **Variable** | **Total (SD)** | **Female (SD)** | **Male (SD)** |
| --- | --- | --- | --- |
| P(Response\|Signal) | 0.30 (0.23) | 0.32 (0.24) | 0.29 (0.22) |
| P(No Response\|Go Trial) | 0.07 (0.06) | 0.07 (0.06) | 0.07 (0.06) |
| P(Choice Error\|Go Trial) | 0.01 (0.01) | 0.01 (0.02) | 0.01 (0.01) |
| *M* RT on Go Trials | 674.39 (82.39) | 667.19 (81.99) | 681.79 (82.43) |
| Intra-subject *SD* for *M* RT on Go Trials | 123.27 (30.78) | 123.57 (28.07) | 122.96 (33.44) |
| Mean Stop Signal Delay | 138.13 (74.99) | 144.57 (78.02) | 131.51 (71.41) |
| Mean RT for Unsuccessful Stop Trials | 536.42 (64.17) | 533.79 (62.90) | 539.21 (65.61) |

## Missing data

**Table S2**

*Percentage of missing data by variable*

| **Variable** | **% Missing** |
| --- | --- |
| Relationship Status | 3.38% |
| Employment | 3.38% |
| Education | 1.35% |
| Household Income | 1.01% |
| Age | 1.01% |
| STRAIN | 0.68% |
| BART | 0.34% |
| 1 - AUC | 1.01% |

*Note.* STRAIN = The Stress and Adversity Inventory for Adults; DERS-SF = Difficulties in Emotional Regulation Scale Short Form; BART = average number of space bar presses for unburst balloons during the Balloon Analogue Risk Task; 1 – AUC = 1 minus the area under the curve scores (greater scores reflect greater delay discounting) for the Titrating Alternatives Delay Discounting Task.

## Exploratory Factor Analysis (EFA)

We randomly split our dataset in half as per our preregistration as we had planned to run EFA on one half of the data and confirmatory factor analysis (CFA) on the other half. However, as the EFA analyses did not achieve simple structure (see below), CFA analysis was not performed.

### Socioeconomic status index

An EFA was used to try to create an index of socioeconomic status using the *psych* package (version 2.1.3) in R version (4.2.1). The variables entered into this EFA included:

1. Highest level of education completed (no formal qualifications, secondary education [GCSE], A-levels, technical/community college, undergraduate degree [BA/BSc/other], graduate degree [MA/MSc/MPhil/other], doctoral degree or higher).
2. Employment status (unemployed, student, employed)
3. Household income (< 10,000, £10,000 - £15,999, £16,000 - £19,999, £20,000 - £29,999, £30,000 - £39,999, £40,000 - £49,999, £50,000 - £59,999, £60,000 - £69,999, £70,000 - £79,999, £80,000 - £89,999, £90,000 - £99,999, £100,000 - £149,999, > £150,000).
4. Subjective social status (1 to 10)

The EFA analysis was conducted using guidelines outlined in Preacher and MacCallum (2003). As that dataset that underwent EFA included a mix of continuous and polytomous variables, the *mixed.cor* function from the *psych* package was used to calculate the correlation matrix that was subjected to EFA (see Table S3). Bartlett’s test indicated correlation adequacy, *ꭓ*^2^ (6) = 42.75, *p* < .001 and the determinant (|*R*| = .733) was well above the specified cut off of .00001, suggesting that the data were not multicollinear. However, the KMO test indicated sampling inadequacy, *MSA* = 0.53. Similarly, individual KMO values were all ‘unacceptable’ (< 0.60) and three methods commonly used to determine how many factors to retain (parallel analysis, scree plot examination, and the K1 criterion) suggested that the data were not suitable for factor analysis (see Figure S1).

**Table S3**

*Inter-correlations of socioeconomic status variables.*

|  | **1** | **2** | **3** |
| --- | --- | --- | --- |
| 1. Education | - |  |  |
| 2. Employment | .23 | - |  |
| 3. Household Income | .12 | .19 | - |
| 4. Subjective Status | .22 | .05 | .40 |

**Figure S1**

*Parallel analysis scree plots.*

*
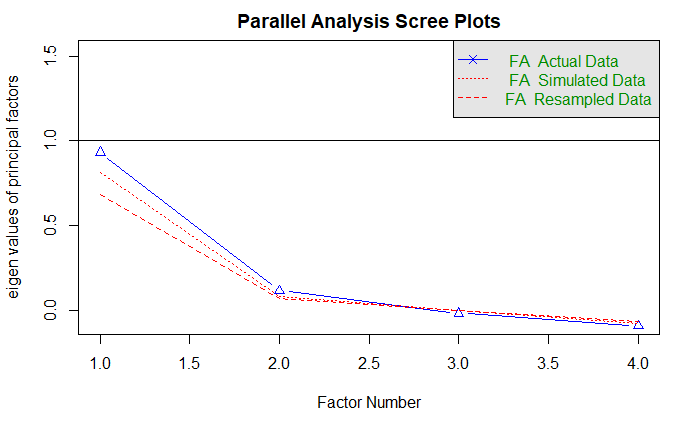
*

### Impulsivity index

The same EFA procedure reported above was followed to try to create a single index of impulsivity. The variables entered into this EFA included:

1. BART (continuous)
2. 1 – AUC (continuous)
3. SSRT (continuous)
4. SUPPS-P Negative Urgency (continuous)
5. SUPPS-P Perseverance (continuous)
6. SUPPS-P Premeditation (continuous)
7. SUPPS-P Sensation Seeking (continuous)
8. SUPPS-P Positive Urgency (continuous)

The correlation matrix that was subjected to EFA is reported in Table S4. Bartlett’s test indicated correlation adequacy, *ꭓ*^2^ (28) = 198.74, *p* < .001, the KMO test indicated sampling adequacy, *MSA* = 0.59, and the determinant (|*R*| = .225) was well above the specified cut off of .00001, suggesting that the data were not multicollinear. However, individual KMO values were ‘unacceptable’ (< .60) for BART, Perseverance, Sensation Seeking and Positive Urgency.

Parallel analysis and scree plot inspection (see Figure S2) suggested that a three-factor model was appropriate. Meanwhile the K1 criterion suggested that one factor solution. Therefore, we tested a three-factor solution. Maximum likelihood was used with direct oblimin rotation. The factor loadings are presented in Table S5. As simple structure was not achieved, we concluded, that like in prior research, impulsivity should be considered as a distinct set of separate constructs (Strickland and Johnson, 2020). Therefore, we estimated separate models, which aimed to test our hypotheses, for each construct.

**Table S4**

*Inter-correlations of impulsivity variables.*

|  | **1** | **2** | **3** | **4** | **5** | **6** | **7** |
| --- | --- | --- | --- | --- | --- | --- | --- |
| 1. BART | - |  |  |  |  |  |  |
| 2. 1 - AUC | -.15 | - |  |  |  |  |  |
| 3. SSRT | .04 | -.02 | - |  |  |  |  |
| 4. SUPPS-P Negative Urgency | -.06 | .15 | -.07 | - |  |  |  |
| 5. SUPPS-P Perseverance | .10 | -.09 | .06 | .22 | - |  |  |
| 6. SUPPS-P Premeditation | .04 | .06 | .00 | .45 | .53 | - |  |
| 7. SUPPS-P Sensation Seeking | .15 | .12 | -.13 | .17 | -.06 | .12 | - |
| 8. SUPPS-P Positive Urgency | -.01 | .14 | -.11 | .63 | .07 | .33 | .44 |

**Figure S2**

*Parallel analysis scree plots.*

*
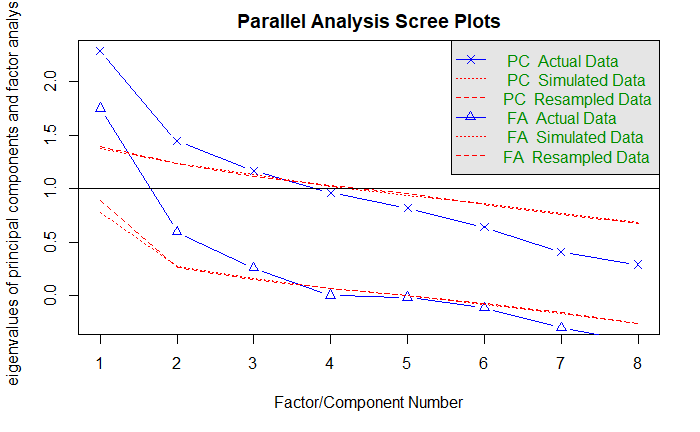
*

**Table S5**

*Three-factor model loadings*

|  | **1** | **2** | **3** |
| --- | --- | --- | --- |
| BART | -.16 | .16 | .21 |
| 1 - AUC | .20 | -.14 | .05 |
| SSRT | -.09 | .08 | -.10 |
| SUPPS-P Negative Urgency | **.90** | .03 | -.09 |
| SUPPS-P Perseverance | -.03 | **.94** | .00 |
| SUPPS-P Premeditation | .35 | **.49** | .04 |
| SUPPS-P Sensation Seeking | .00 | .00 | **1.00** |
| SUPPS-P Positive Urgency | **.69** | -.06 | .24 |

*Boldface font indicates factor loadings > .04.*

## Lifetime alcohol use descriptive statistics

**Figure S3**

*Descriptive statistics for lifetime alcohol use in terms of average units of alcohol consumed per week (A), the type of beverage consumed (B), the time of drinking (C), the time of drinking, and drinking context (D).* *1 UK alcohol unit = 8g of pure ethanol.*

*
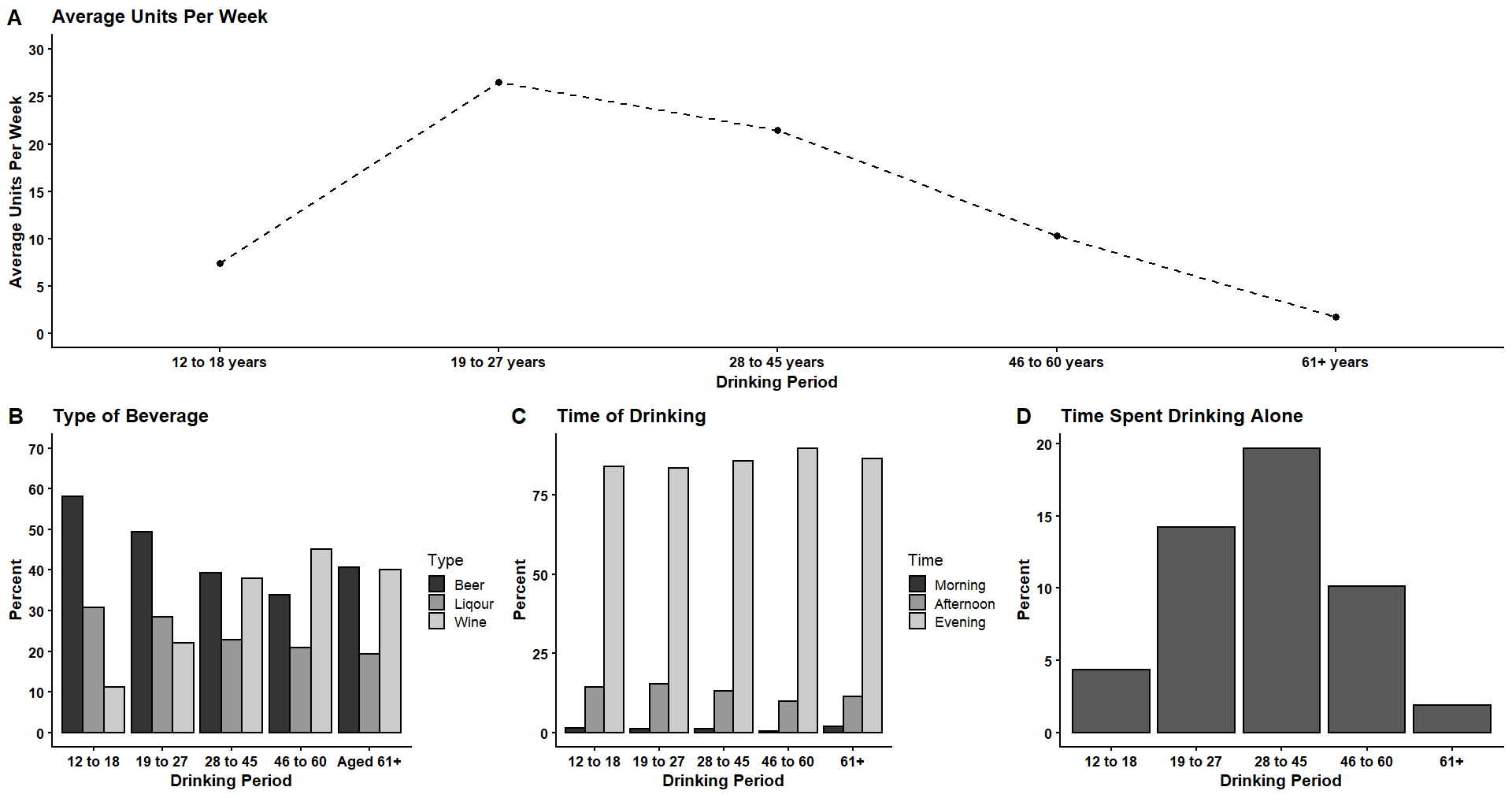
*

## Bivariate analyses

**Table S6**

*Inter-correlations (Spearman’s rank values) of key study variables*

|  | **1** | **2** | **3** | **4** | **5** | **6** | **7** | **8** | **9** | **10** | **11** |
| --- | --- | --- | --- | --- | --- | --- | --- | --- | --- | --- | --- |
| 1. Stressor Count (STRAIN) | - |  |  |  |  |  |  |  |  |  |  |
| 2. Stressor Severity (STRAIN) | .92^**^ | - |  |  |  |  |  |  |  |  |  |
| 3. DERS-SF | .24^**^ | .28^**^ | - |  |  |  |  |  |  |  |  |
| 4. AUDIT | .32^**^ | .34^**^ | .41^**^ | - |  |  |  |  |  |  |  |
| 5. Weekly Consumption (Units) | .22^**^ | .26^**^ | .24^**^ | .69^**^ | - |  |  |  |  |  |  |
| 6. SUPPS-P Negative Urgency | .21^**^ | .25^**^ | .63^**^ | .38^**^ | .24^**^ | - |  |  |  |  |  |
| 7. SUPPS-P Perseverance | .08 | .06 | .21^**^ | .16^**^ | .00 | .13^*^ | - |  |  |  |  |
| 8. SUPPS-P Premeditation | .19^**^ | .22^**^ | .33^**^ | .30^**^ | .15^*^ | .34^**^ | .44^**^ | - |  |  |  |
| 9. SUPPS-P Sensation Seeking | -.05 | -.09 | .09 | .06 | .03 | .18^**^ | -.17^**^ | .06 | - |  |  |
| 10. SUPPS-P Positive Urgency | .12^*^ | .14^*^ | .50^**^ | .41^**^ | .28^**^ | .68^**^ | .09 | .36^**^ | .39^**^ | - |  |
| 11. BART | .06 | .01 | -.08 | .02 | .02 | -.04 | .05 | .00 | .12^*^ | .03 | - |
| 12. 1 - AUC | .03 | .05 | .07 | .07 | .13^*^ | .15^*^ | -.05 | .11 | .01 | .11 | -.12^*^ |

*Note.* STRAIN = Stress and Adversity Inventory for Adults; DERS-SF = Difficulties in Emotional Regulation Scale Short Form; AUDIT = Alcohol Use Disorders Identification Test; 1 unit = 8g pure ethanol; SUPPS-P = Shortened Urgency, Premeditation (lack of), Perseverance (lack of), Sensation Seeking, Positive Urgency, Impulsive Behaviour Scale; BART = average number of space bar presses for unburst balloons during the Balloon Analogue Risk Task; 1 – AUC = 1 minus the area under the curve scores (greater scores reflect greater delay discounting) for the Titrating Alternatives Delay Discounting Task.

* *p* < .05, ** *p* < .01, *** *p* < .001.

## Conditional process analysis (i.e., moderated mediation analysis)

**Table S7**

*Summary of the mediation analysis examining whether negative urgency moderates the mediated relation between cumulative lifetime stressor exposure, emotional dysregulation and lifetime alcohol use (N = 279)*

|  |  | **Consequent** | | | | | | | | |
| --- | --- | --- | --- | --- | --- | --- | --- | --- | --- | --- |
|  |  | ***M* (DERS-SF)** | | | |  | ***Y* (Alcohol Use)** | | | |
| **Antecedent** |  | **B** | **SE** | **LL** | **UL** |  | **B** | **SE** | **LL** | **UL** |
| Constant | *i_M_* | 26.00 | 5.88 | 14.56 | 37.52 | *i_Y_* | 72.88 | 35.41 | 10.16 | 146.74 |
| *X* (STRAIN) | *a* | **-0.15** | **0.07** | **-0.29** | **-0.02** | *c'* | 0.23 | 0.25 | -0.28 | 0.70 |
| *M* (DERS-SF) |  | - | - | - | - | *b_1_* | -1.08 | 0.62 | -2.29 | 0.12 |
| *W* (NEGURG) |  | **1.48** | **0.37** | **0.76** | **2.20** | *b_2_* | -3.70 | 2.65 | -9.01 | 1.47 |
| *W* x *X* |  | **0.02** | **0.01** | **0.01** | **0.03** | *b_3_* | -0.01 | 0.02 | -0.06 | 0.03 |
| *W* x *M* |  | - | - | - | - | *b_4_* | **0.13** | **0.07** | **0.005** | **0.26** |
| Age |  | **-0.17** | **0.05** | **-0.28** | **-0.06** |  | -0.06 | 0.16 | -0.39 | 0.26 |
| Sex = Male |  | 1.13 | 1.15 | -1.20 | 3.38 |  | 7.72 | 3.97 | -0.16 | 15.50 |
| Education |  |  |  |  |  |  |  |  |  |  |
| GCSE & below |  | Ref. |  |  |  |  | Ref. |  |  |  |
| A-levels & equivalent |  | 4.26 | 2.39 | -0.30 | 9.12 |  | -13.73 | 9.91 | -34.39 | 4.89 |
| Undergraduate & higher |  | 2.85 | 2.11 | -1.17 | 7.07 |  | -15.94 | 9.58 | -35.90 | 1.67 |
| Employment |  |  |  |  |  |  |  |  |  |  |
| Unemployed |  | Ref. |  |  |  |  | Ref. |  |  |  |
| Student |  | 5.73 | 3.03 | -0.35 | 11.58 |  | -6.69 | 15.89 | -40.31 | 20.23 |
| Employed |  | 4.23 | 2.47 | -0.72 | 8.99 |  | -11.58 | 13.92 | -41.47 | 10.33 |
| Household Income |  |  |  |  |  |  |  |  |  |  |
| Low |  | Ref. |  |  |  |  | Ref. |  |  |  |
| Medium |  | 0.47 | 2.04 | -3.48 | 4.54 |  | 0.29 | 5.01 | -9.07 | 10.59 |
| High |  | -1.45 | 1.41 | -4.16 | 1.36 |  | -0.10 | 5.09 | -9.62 | 10.52 |
| Subjective Social Status |  |  |  |  |  |  |  |  |  |  |
| Low |  | Ref. |  |  |  |  | Ref. |  |  |  |
| Medium |  | -0.13 | 1.74 | -3.59 | 3.25 |  | 3.10 | 6.69 | -8.80 | 17.38 |
| High |  | -1.19 | 1.51 | -4.18 | 1.74 |  | -0.42 | 4.56 | -9.16 | 8.60 |
|  |  |  |  |  |  |  |  |  |  |  |
|  |  | R^2^ = 0.51 | | | |  | R^2^ = 0.14 | | | |
|  |  | *F*(13, 265) = 21.53, *p* < .001 | | | |  | *F*(15, 263) = 2.90, *p* < .001 | | | |

*Note.* Models were adjusted for age, sex, highest level of education achieved, employment status, and household income. LL and UL represent the lower and upper limit of the bootstrapped 95% CI (10,000 bootstraps), respectively. STRAIN = Stress and Adversity Inventory for Adults Stressor Severity Index; DERS-SF = Difficulties in Emotional Regulation Scale Short Form; NEGURG = Negative Urgency subscale of the Shortened Urgency, Premeditation, Perseverance, Sensation Seeking, Positive Urgency, Impulsive Behaviour Scale; 1 unit = 8g pure ethanol. Significant effects (*p* < .05) are in boldface.

**Table S8**

*Summary of the mediation analysis examining whether lack of perseverance moderates the mediated relation between cumulative lifetime stressor exposure, emotional dysregulation and lifetime alcohol use (N = 279)*

|  |  | **Consequent** | | | | | | | | |
| --- | --- | --- | --- | --- | --- | --- | --- | --- | --- | --- |
|  |  | ***M* (DERS-SF)** | | | |  | ***Y* (Alcohol Use)** | | | |
| **Antecedent** |  | **B** | **SE** | **LL** | **UL** |  | **B** | **SE** | **LL** | **UL** |
| Constant | *i_M_* | **34.66** | **7.53** | **19.86** | **49.30** | *i_Y_* | **114.43** | **40.08** | **40.98** | **195.38** |
| *X* (STRAIN) | *a* | 0.12 | 0.11 | -0.08 | 0.34 | *c'* | -0.21 | 0.29 | -0.83 | 0.33 |
| *M* (DERS-SF) |  | - | - | - | - | *b_1_* | -1.02 | 0.62 | -2.15 | 0.27 |
| *W* (PERSEV) |  | 1.23 | 0.67 | -0.05 | 2.57 | *b_2_* | **-11.89** | **4.00** | **-19.62** | **-3.84** |
| *W* x *X* |  | 0.003 | 0.01 | -0.03 | 0.03 | *b_3_* | 0.04 | 0.04 | -0.03 | 0.12 |
| *W* x *M* |  | - | - | - | - | *b_4_* | **0.21** | **0.09** | **0.02** | **0.37** |
| Age |  | **-0.34** | **0.06** | **-0.45** | **-0.22** |  | -0.01 | 0.17 | -0.34 | 0.30 |
| Sex = Male |  | -0.56 | 1.43 | -3.42 | 2.26 |  | 6.70 | 3.87 | -0.90 | 14.30 |
| Education |  |  |  |  |  |  |  |  |  |  |
| GCSE & below |  | Ref. |  |  |  |  | Ref. |  |  |  |
| A-levels & equivalent |  | 4.24 | 3.10 | -1.87 | 10.25 |  | -16.07 | 9.62 | -36.00 | 1.61 |
| Undergraduate & higher |  | 2.21 | 2.87 | -3.44 | 7.87 |  | -16.70 | 9.41 | -36.47 | 0.30 |
| Employment |  |  |  |  |  |  |  |  |  |  |
| Unemployed |  | Ref. |  |  |  |  | Ref. |  |  |  |
| Student |  | 6.37 | 3.72 | -1.21 | 13.52 |  | -5.67 | 14.93 | -37.01 | 20.37 |
| Employed |  | 5.57 | 2.95 | -0.52 | 11.08 |  | -12.15 | 13.83 | -41.51 | 11.17 |
| Household Income |  |  |  |  |  |  |  |  |  |  |
| Low |  | Ref. |  |  |  |  | Ref. |  |  |  |
| Medium |  | -1.44 | 2.21 | -5.55 | 3.08 |  | -1.23 | 4.82 | -10.12 | 8.85 |
| High |  | -2.03 | 1.80 | -5.46 | 1.68 |  | -0.07 | 4.97 | -9.39 | 10.14 |
| Subjective Social Status |  |  |  |  |  |  |  |  |  |  |
| Low |  | Ref. |  |  |  |  | Ref. |  |  |  |
| Medium |  | -0.52 | 2.07 | -4.69 | 3.42 |  | 2.50 | 6.62 | -9.16 | 16.85 |
| High |  | -1.00 | 1.89 | -4.77 | 2.71 |  | -0.47 | 4.33 | -8.76 | 8.20 |
|  |  |  |  |  |  |  |  |  |  |  |
|  |  | R^2^ = 0.27 | | | |  | R^2^ = 0.16 | | | |
|  |  | *F*(13, 265) = 7.54, *p* < .001 | | | |  | *F*(15, 263) = 3.30, *p* < .001 | | | |

*Note.* Models were adjusted for age, sex, highest level of education achieved, employment status, and household income. LL and UL represent the lower and upper limit of the bootstrapped 95% CI (10,000 bootstraps), respectively. STRAIN = Stress and Adversity Inventory for Adults Stressor Severity Index; DERS-SF = Difficulties in Emotional Regulation Scale Short Form; PERSEV = Perseverance subscale of the Shortened Urgency, Premeditation, Perseverance, Sensation Seeking, Positive Urgency, Impulsive Behaviour Scale; 1 unit = 8g pure ethanol. Significant effects (*p* < .05) are in boldface.

**Table S9**

*Summary of the mediation analysis examining whether lack of premeditation moderates the mediated relation between cumulative lifetime stressor exposure, emotional dysregulation and lifetime alcohol use (N = 279)*

|  |  | **Consequent** | | | | | | | | |
| --- | --- | --- | --- | --- | --- | --- | --- | --- | --- | --- |
|  |  | ***M* (DERS-SF)** | | | |  | ***Y* (Alcohol Use)** | | | |
| **Antecedent** |  | **B** | **SE** | **LL** | **UL** |  | **B** | **SE** | **LL** | **UL** |
| Constant | *i_M_* | **31.43** | **6.46** | **19.30** | **44.48** | *i_Y_* | **69.05** | **34.66** | **5.96** | **140.47** |
| *X* (STRAIN) | *a* | 0.003 | 0.08 | -0.14 | 0.16 | *c'* | 0.37 | 0.27 | -0.24 | 0.84 |
| *M* (DERS-SF) |  | - | - | - | - | *b_1_* | -0.79 | 0.60 | -1.85 | 0.53 |
| *W* (PREMED) |  | **1.24** | **0.55** | **0.16** | **2.32** | *b_2_* | -5.38 | 3.67 | -12.29 | 2.20 |
| *W* x *X* |  | 0.01 | 0.01 | -0.01 | 0.03 | *b_3_* | -0.04 | 0.04 | -0.10 | 0.05 |
| *W* x *M* |  | - | - | - | - | *b_4_* | 0.17 | 0.09 | -0.03 | 0.34 |
| Age |  | **-0.29** | **0.06** | **-0.41** | **-0.17** |  | -0.06 | 0.17 | -0.40 | 0.25 |
| Sex = Male |  | 0.17 | 1.38 | -2.53 | 2.93 |  | 6.73 | 3.96 | -1.25 | 14.43 |
| Education |  |  |  |  |  |  |  |  |  |  |
| GCSE & below |  | Ref. |  |  |  |  | Ref. |  |  |  |
| A-levels & equivalent |  | 4.48 | 3.07 | -1.63 | 10.48 |  | -16.24 | 10.13 | -37.54 | 2.34 |
| Undergraduate & higher |  | 3.21 | 2.77 | -2.31 | 8.58 |  | -18.22 | 9.86 | -39.06 | -0.09 |
| Employment |  |  |  |  |  |  |  |  |  |  |
| Unemployed |  | Ref. |  |  |  |  | Ref. |  |  |  |
| Student |  | **8.67** | **3.33** | **1.82** | **14.88** |  | -5.24 | 16.08 | -39.65 | 22.28 |
| Employed |  | **7.74** | **2.56** | **2.51** | **12.64** |  | -11.80 | 14.69 | -43.97 | 11.88 |
| Household Income |  |  |  |  |  |  |  |  |  |  |
| Low |  | Ref. |  |  |  |  | Ref. |  |  |  |
| Medium |  | -1.86 | 2.35 | -6.30 | 2.91 |  | -0.71 | 5.20 | -10.54 | 9.84 |
| High |  | -2.49 | 1.70 | -5.71 | 0.96 |  | 0.24 | 5.17 | -9.47 | 10.88 |
| Subjective Social Status |  |  |  |  |  |  |  |  |  |  |
| Low |  | Ref. |  |  |  |  | Ref. |  |  |  |
| Medium |  | -1.06 | 2.05 | -5.15 | 2.86 |  | 3.17 | 6.69 | -8.82 | 17.54 |
| High |  | -1.05 | 1.80 | -4.55 | 2.45 |  | 0.64 | 4.54 | -8.06 | 9.74 |
|  |  |  |  |  |  |  |  |  |  |  |
|  |  | R^2^ = 0.32 | | | |  | R^2^ = 0.13 | | | |
|  |  | *F*(13, 265) = 9.79, *p* < .001 | | | |  | *F*(15, 263) = 2.73, *p* < .001 | | | |

*Note.* Models were adjusted for age, sex, highest level of education achieved, employment status, and household income. LL and UL represent the lower and upper limit of the bootstrapped 95% CI (10,000 bootstraps), respectively. STRAIN = Stress and Adversity Inventory for Adults Stressor Severity Index; DERS-SF = Difficulties in Emotional Regulation Scale Short Form; PREMED = Premeditation subscale of the Shortened Urgency, Premeditation, Perseverance, Sensation Seeking, Positive Urgency, Impulsive Behaviour Scale; 1 unit = 8g pure ethanol. Significant effects (*p* < .05) are in boldface.

**Table S10**

*Summary of the mediation analysis examining whether sensation seeking moderates the mediated relation between cumulative lifetime stressor exposure, emotional dysregulation and lifetime alcohol use (N = 279)*

|  |  | **Consequent** | | | | | | | | |
| --- | --- | --- | --- | --- | --- | --- | --- | --- | --- | --- |
|  |  | ***M* (DERS-SF)** | | | |  | ***Y* (Alcohol Use)** | | | |
| **Antecedent** |  | **B** | **SE** | **LL** | **UL** |  | **B** | **SE** | **LL** | **UL** |
| Constant | *i_M_* | **42.83** | **7.50** | **27.78** | **57.37** | *i_Y_* | 5.59 | 30.99 | -49.11 | 72.03 |
| *X* (STRAIN) | *a* | 0.11 | 0.10 | -0.09 | 0.31 | *c'* | 0.49 | 0.26 | -0.02 | 0.99 |
| *M* (DERS-SF) |  | - | - | - | - | *b_1_* | 1.02 | 0.64 | -0.35 | 2.16 |
| *W* (SENSAT) |  | 0.05 | 0.58 | -1.03 | 1.23 | *b_2_* | 3.30 | 2.54 | -2.00 | 7.90 |
| *W* x *X* |  | 0.004 | 0.01 | -0.02 | 0.02 | *b_3_* | -0.04 | 0.02 | -0.08 | 0.01 |
| *W* x *M* |  | - | - | - | - | *b_4_* | -0.05 | 0.06 | -0.16 | 0.07 |
| Age |  | **-0.32** | **0.06** | **-0.44** | **-0.20** |  | -0.11 | 0.17 | -0.46 | 0.21 |
| Sex = Male |  | -0.76 | 1.55 | -3.89 | 2.25 |  | 6.55 | 3.99 | -1.73 | 14.16 |
| Education |  |  |  |  |  |  |  |  |  |  |
| GCSE & below |  | Ref. |  |  |  |  | Ref. |  |  |  |
| A-levels & equivalent |  | 4.85 | 3.28 | -1.69 | 11.32 |  | **-20.17** | **9.37** | **-39.18** | **-2.44** |
| Undergraduate & higher |  | 2.89 | 3.03 | -3.14 | 8.78 |  | **-22.03** | **9.22** | **-40.97** | **-4.63** |
| Employment |  |  |  |  |  |  |  |  |  |  |
| Unemployed |  | Ref. |  |  |  |  | Ref. |  |  |  |
| Student |  | 5.60 | 3.84 | -2.16 | 12.99 |  | -7.52 | 17.14 | -45.23 | 19.29 |
| Employed |  | 5.16 | 3.06 | -1.09 | 10.95 |  | -14.54 | 15.67 | -50.02 | 8.11 |
| Household Income |  |  |  |  |  |  |  |  |  |  |
| Low |  | Ref. |  |  |  |  | Ref. |  |  |  |
| Medium |  | -1.24 | 2.27 | -5.45 | 3.36 |  | -2.74 | 5.15 | -12.39 | 7.72 |
| High |  | -1.63 | 1.84 | -5.15 | 2.12 |  | -1.25 | 5.35 | -11.24 | 9.80 |
| Subjective Social Status |  |  |  |  |  |  |  |  |  |  |
| Low |  | Ref. |  |  |  |  | Ref. |  |  |  |
| Medium |  | -0.99 | 2.19 | -5.43 | 3.16 |  | 3.02 | 6.72 | -9.03 | 17.25 |
| High |  | -2.15 | 1.91 | -5.94 | 1.63 |  | 0.63 | 4.68 | -8.46 | 9.78 |
|  |  |  |  |  |  |  |  |  |  |  |
|  |  | R^2^ = 0.23 | | | |  | R^2^ = 0.13 | | | |
|  |  | *F*(13, 265) = 6.11, *p* < .001 | | | |  | *F*(15, 263) = 2.54, *p* < .001 | | | |

*Note.* Models were adjusted for age, sex, highest level of education achieved, employment status, and household income. LL and UL represent the lower and upper limit of the bootstrapped 95% CI (10,000 bootstraps), respectively. STRAIN = Stress and Adversity Inventory for Adults Stressor Severity Index; DERS-SF = Difficulties in Emotional Regulation Scale Short Form; SENSAT = Sensation Seeking subscale of the Shortened Urgency, Premeditation, Perseverance, Sensation Seeking, Positive Urgency, Impulsive Behaviour Scale; 1 unit = 8g pure ethanol. Significant effects (*p* < .05) are in boldface.

**Table S11**

*Summary of the mediation analysis examining whether positive urgency moderates the mediated relation between cumulative lifetime stressor exposure, emotional dysregulation and lifetime alcohol use (N = 279)*

|  |  | **Consequent** | | | | | | | | |
| --- | --- | --- | --- | --- | --- | --- | --- | --- | --- | --- |
|  |  | ***M* (DERS-SF)** | | | |  | ***Y* (Alcohol Use)** | | | |
| **Antecedent** |  | **B** | **SE** | **LL** | **UL** |  | **B** | **SE** | **LL** | **UL** |
| Constant | *i_M_* | **29.21** | **6.49** | **16.79** | **42.27** | *i_Y_* | 26.48 | 23.14 | -15.12 | 75.17 |
| *X* (STRAIN) | *a* | 0.08 | 0.07 | -0.07 | 0.22 | *c'* | **0.54** | **0.22** | **0.12** | **1.00** |
| *M* (DERS-SF) |  | - | - | - | - | *b_1_* | 0.14 | 0.48 | -0.82 | 1.04 |
| *W* (POSURG) |  | **1.67** | **0.52** | **0.66** | **2.72** | *b_2_* | 1.19 | 2.38 | -3.62 | 5.77 |
| *W* x *X* |  | 0.004 | 0.01 | -0.01 | 0.02 | *b_3_* | **-0.05** | **0.03** | **-0.10** | **-0.001** |
| *W* x *M* |  | - | - | - | - | *b_4_* | 0.04 | 0.05 | -0.06 | 0.15 |
| Age |  | **-0.23** | **0.06** | **-0.35** | **-0.12** |  | -0.05 | 0.17 | -0.38 | 0.28 |
| Sex = Male |  | -0.74 | 1.32 | -3.34 | 1.84 |  | 6.75 | 3.97 | -1.30 | 14.39 |
| Education |  |  |  |  |  |  |  |  |  |  |
| GCSE & below |  | Ref. |  |  |  |  | Ref. |  |  |  |
| A-levels & equivalent |  | 4.02 | 3.05 | -1.91 | 10.03 |  | **-17.86** | **9.51** | **-37.89** | **-0.24** |
| Undergraduate & higher |  | 3.44 | 2.75 | -1.98 | 8.87 |  | **-19.19** | **9.32** | **-38.87** | **-2.06** |
| Employment |  |  |  |  |  |  |  |  |  |  |
| Unemployed |  | Ref. |  |  |  |  | Ref. |  |  |  |
| Student |  | 4.68 | 3.76 | -2.96 | 11.74 |  | -9.50 | 17.90 | -47.93 | 19.59 |
| Employed |  | 4.40 | 3.01 | -1.91 | 9.94 |  | -15.33 | 16.43 | -51.50 | 9.22 |
| Household Income |  |  |  |  |  |  |  |  |  |  |
| Low |  | Ref. |  |  |  |  | Ref. |  |  |  |
| Medium |  | -0.16 | 2.24 | -4.43 | 4.37 |  | -0.58 | 4.83 | -9.79 | 9.16 |
| High |  | -2.12 | 1.58 | -5.12 | 1.11 |  | -0.74 | 5.20 | -10.46 | 9.92 |
| Subjective Social Status |  |  |  |  |  |  |  |  |  |  |
| Low |  | Ref. |  |  |  |  | Ref. |  |  |  |
| Medium |  | -1.12 | 2.07 | -5.25 | 2.86 |  | 2.69 | 6.64 | -9.19 | 16.68 |
| High |  | -1.73 | 1.81 | -5.25 | 1.89 |  | -0.38 | 4.66 | -9.34 | 8.78 |
|  |  |  |  |  |  |  |  |  |  |  |
|  |  | R^2^ = 0.36 | | | |  | R^2^ = 0.12 | | | |
|  |  | *F*(13, 265) = 11.29, *p* < .001 | | | |  | *F*(15, 263) = 2.40, *p* < .001 | | | |

*Note.* Models were adjusted for age, sex, highest level of education achieved, employment status, and household income. LL and UL represent the lower and upper limit of the bootstrapped 95% CI (10,000 bootstraps), respectively. STRAIN = Stress and Adversity Inventory for Adults Stressor Severity Index; DERS-SF = Difficulties in Emotional Regulation Scale Short Form; POSURG = Positive Urgency subscale of the Shortened Urgency, Premeditation, Perseverance, Sensation Seeking, Positive Urgency, Impulsive Behaviour Scale; 1 unit = 8g pure ethanol. Significant effects (*p* < .05) are in boldface.

**Table S12**

*Summary of the mediation analysis examining whether risk-taking (BART scores) moderates the mediated relation between cumulative lifetime stressor exposure, emotional dysregulation and lifetime alcohol use (N = 279)*

|  |  | **Consequent** | | | | | | | | |
| --- | --- | --- | --- | --- | --- | --- | --- | --- | --- | --- |
|  |  | ***M* (DERS-SF)** | | | |  | ***Y* (Alcohol Use)** | | | |
| **Antecedent** |  | **B** | **SE** | **LL** | **UL** |  | **B** | **SE** | **LL** | **UL** |
| Constant | *i_M_* | **47.63** | **6.61** | **34.79** | **60.88** | *i_Y_* | 30.07 | 25.63 | -16.26 | 82.75 |
| *X* (STRAIN) | *a* | 0.12 | 0.07 | -0.03 | 0.26 | *c'* | 0.07 | 0.21 | -0.36 | 0.46 |
| *M* (DERS-SF) |  | - | - | - | - | *b_1_* | 0.57 | 0.39 | -0.25 | 1.30 |
| *W* (BART) |  | -0.10 | 0.11 | -0.33 | 0.11 | *b_2_* | 0.07 | 0.55 | -1.09 | 1.08 |
| *W* x *X* |  | 0.001 | 0.002 | -0.003 | 0.01 | *b_3_* | 0.002 | 0.01 | -0.01 | 0.02 |
| *W* x *M* |  | - | - | - | - | *b_4_* | -0.003 | 0.01 | -0.02 | 0.02 |
| Age |  | **-0.33** | **0.06** | **-0.44** | **-0.21** |  | -0.06 | 0.17 | -0.40 | 0.27 |
| Sex = Male |  | -0.73 | 1.46 | -3.62 | 2.14 |  | 6.93 | 3.95 | -0.79 | 14.58 |
| Education |  |  |  |  |  |  |  |  |  |  |
| GCSE & below |  | Ref. |  |  |  |  | Ref. |  |  |  |
| A-levels & equivalent |  | 3.66 | 3.33 | -2.84 | 10.19 |  | -17.53 | 9.80 | -37.77 | 0.73 |
| Undergraduate & higher |  | 1.87 | 3.12 | -4.37 | 7.97 |  | **-19.16** | **9.42** | **-39.01** | **-1.93** |
| Employment |  |  |  |  |  |  |  |  |  |  |
| Unemployed |  | Ref. |  |  |  |  | Ref. |  |  |  |
| Student |  | 5.83 | 3.83 | -2.05 | 13.06 |  | -5.96 | 17.74 | -43.93 | 23.58 |
| Employed |  | 4.89 | 2.98 | -1.29 | 10.38 |  | -12.12 | 15.99 | -47.38 | 12.02 |
| Household Income |  |  |  |  |  |  |  |  |  |  |
| Low |  | Ref. |  |  |  |  | Ref. |  |  |  |
| Medium |  | -1.01 | 2.17 | -5.29 | 3.25 |  | -2.22 | 4.94 | -11.78 | 7.84 |
| High |  | -1.23 | 1.81 | -4.75 | 2.37 |  | -0.08 | 5.04 | -9.25 | 10.60 |
| Subjective Social Status |  |  |  |  |  |  |  |  |  |  |
| Low |  | Ref. |  |  |  |  | Ref. |  |  |  |
| Medium |  | -1.31 | 2.14 | -5.63 | 2.85 |  | 2.58 | 6.61 | -9.72 | 16.43 |
| High |  | -2.32 | 1.94 | -6.02 | 1.44 |  | 0.10 | 4.80 | -9.26 | 9.71 |
|  |  |  |  |  |  |  |  |  |  |  |
|  |  | R^2^ = 0.23 | | | |  | R^2^ = 0.11 | | | |
|  |  | *F*(13, 264) = 6.11, *p* < .001 | | | |  | *F*(15, 262) = 2.08, *p* = 0.011 | | | |

*Note.* Models were adjusted for age, sex, highest level of education achieved, employment status, and household income. LL and UL represent the lower and upper limit of the bootstrapped 95% CI (10,000 bootstraps), respectively. STRAIN = Stress and Adversity Inventory for Adults Stressor Severity Index; DERS-SF = Difficulties in Emotional Regulation Scale Short Form; BART = average number of space bar presses for unburst balloons during the Balloon Analogue Risk Task; 1 unit = 8g pure ethanol. Significant effects (*p* < .05) are in boldface.

**Table S13**

*Summary of the mediation analysis examining whether delay discounting (1 - AUC) moderates the mediated relation between cumulative lifetime stressor exposure, emotional dysregulation and lifetime alcohol use (N = 279)*

|  |  | **Consequent** | | | | | | | | |
| --- | --- | --- | --- | --- | --- | --- | --- | --- | --- | --- |
|  |  | ***M* (DERS-SF)** | | | |  | ***Y* (Alcohol Use)** | | | |
| **Antecedent** |  | **B** | **SE** | **LL** | **UL** |  | **B** | **SE** | **LL** | **UL** |
| Constant | *i_M_* | **49.42** | **11.09** | **26.83** | **70.46** | *i_Y_* | 17.30 | 56.74 | -94.50 | 131.25 |
| *X* (STRAIN) | *a* | -0.02 | 0.18 | -0.35 | 0.36 | *c'* | 0.32 | 0.49 | -0.59 | 1.37 |
| *M* (DERS-SF) |  | - | - | - | - | *b_1_* | 0.25 | 1.13 | -1.92 | 2.52 |
| *W* (1 - AUC) |  | -6.29 | 12.23 | -28.91 | 19.59 | *b_2_* | 16.30 | 66.99 | -112.67 | 150.60 |
| *W* x *X* |  | 0.20 | 0.227 | -0.29 | 0.62 | *b_3_* | -0.25 | 0.61 | -1.53 | 0.90 |
| *W* x *M* |  | - | - | - | - | *b_4_* | 0.30 | 1.50 | -2.74 | 3.17 |
| Age |  | **-0.34** | **0.06** | **-0.45** | **-0.22** |  | -0.02 | 0.18 | -0.38 | 0.32 |
| Sex = Male |  | -0.75 | 1.47 | -3.60 | 2.10 |  | 7.01 | 3.95 | -0.78 | 14.75 |
| Education |  |  |  |  |  |  |  |  |  |  |
| GCSE & below |  | Ref. |  |  |  |  | Ref. |  |  |  |
| A-levels & equivalent |  | 3.96 | 3.41 | -2.72 | 10.77 |  | -17.04 | 9.93 | -37.95 | 1.35 |
| Undergraduate & higher |  | 1.70 | 3.19 | -4.62 | 7.93 |  | **-18.57** | **9.56** | **-38.90** | **-0.89** |
| Employment |  |  |  |  |  |  |  |  |  |  |
| Unemployed |  | Ref. |  |  |  |  | Ref. |  |  |  |
| Student |  | 6.10 | 3.90 | -2.07 | 13.21 |  | -6.02 | 18.62 | -47.06 | 23.78 |
| Employed |  | 5.68 | 3.08 | -0.83 | 11.24 |  | -12.02 | 16.93 | -50.33 | 12.93 |
| Household Income |  |  |  |  |  |  |  |  |  |  |
| Low |  | Ref. |  |  |  |  | Ref. |  |  |  |
| Medium |  | -1.23 | 2.22 | -5.59 | 3.25 |  | -1.97 | 5.02 | -11.40 | 8.36 |
| High |  | -0.98 | 1.80 | -4.40 | 2.59 |  | 0.32 | 5.09 | -9.37 | 10.70 |
| Subjective Social Status |  |  |  |  |  |  |  |  |  |  |
| Low |  | Ref. |  |  |  |  | Ref. |  |  |  |
| Medium |  | -0.91 | 2.24 | -5.33 | 3.40 |  | 2.12 | 6.84 | -10.42 | 16.47 |
| High |  | -1.86 | 2.00 | -5.85 | 1.98 |  | -0.31 | 5.22 | -10.38 | 9.89 |
|  |  |  |  |  |  |  |  |  |  |  |
|  |  | R^2^ = 0.22 | | | |  | R^2^ = 0.11 | | | |
|  |  | *F*(13, 262) = 5.71, *p* < .001 | | | |  | *F*(15, 260) = 2.05, *p* = 0.013 | | | |

*Note.* Models were adjusted for age, sex, highest level of education achieved, employment status, and household income. LL and UL represent the lower and upper limit of the bootstrapped 95% CI (10,000 bootstraps), respectively. STRAIN = Stress and Adversity Inventory for Adults Stressor Severity Index; DERS-SF = Difficulties in Emotional Regulation Scale Short Form; 1 – AUC = 1 minus the area under the curve scores (greater scores reflect greater delay discounting) for the Titrating Alternatives Delay Discounting Task; 1 unit = 8g pure ethanol. Significant effects (*p* < .05) are in boldface.

# Supplementary References

Abravanel, B.T., Sinha, R., 2015. Emotion dysregulation mediates the relationship between lifetime cumulative adversity and depressive symptomatology. J. Psychiatr. Res. 61, 89–96. https://doi.org/10.1016/j.jpsychires.2014.11.012

Aurora, P., Klanecky, A.K., 2016. Drinking motives mediate emotion regulation difficulties and problem drinking in college students. Am. J. Drug Alcohol Abuse 42, 341–350. https://doi.org/10.3109/00952990.2015.1133633

Band, G.P.H., van der Molen, M.W., Logan, G.D., 2003. Horse-race model simulations of the stop-signal procedure. Acta Psychol. (Amst.) 112, 105–142. https://doi.org/10.1016/S0001-6918(02)00079-3

Burns, E.E., Jackson, J.L., Harding, H.G., 2010. Child Maltreatment, Emotion Regulation, and Posttraumatic Stress: The Impact of Emotional Abuse. J. Aggress. Maltreatment Trauma 19, 801–819. https://doi.org/10.1080/10926771.2010.522947

Congdon, E., Mumford, J.A., Cohen, J.R., Galvan, A., Canli, T., Poldrack, R.A., 2012. Measurement and Reliability of Response Inhibition. Front. Psychol. 3. https://doi.org/10.3389/fpsyg.2012.00037

Dawson, D.A., Grant, B.F., Ruan, W.J., 2005. The association between stress and drinking: modifying effects of gender and vulnerability. Alcohol Alcohol 40, 453–460. https://doi.org/10.1093/alcalc/agh176

Dragan, M., 2015. Difficulties in emotion regulation and problem drinking in young women: The mediating effect of metacognitions about alcohol use. Addict. Behav. 48, 30–35. https://doi.org/10.1016/j.addbeh.2015.04.008

Fox, H.C., Bergquist, K.L., Gu, P., Sinha, R., 2010. Interactive effects of cumulative stress and impulsivity on alcohol consumption. Alcohol. Clin. Exp. Res. 34, 1376–1385. https://doi.org/10.1111/j.1530-0277.2010.01221.x

Khosravani, V., Sharifi Bastan, F., Ghorbani, F., Kamali, Z., 2017. Difficulties in emotion regulation mediate negative and positive affects and craving in alcoholic patients. Addict. Behav. 71, 75–81. https://doi.org/10.1016/j.addbeh.2017.02.029

Lakens, D., 2022. Sample Size Justification. Collabra Psychol. 8, 33267. https://doi.org/10.1525/collabra.33267

Lappin, J.S., Eriksen, C.W., 1966. Use of a delayed signal to stop a visual reaction-time response. J. Exp. Psychol. 72, 805–811. https://doi.org/10.1037/h0021266

Logan, G.D., Cowan, W.B., 1984. On the ability to inhibit thought and action: A theory of an act of control. Psychol. Rev. 91, 295–327. https://doi.org/10.1037/0033-295X.91.3.295

Mandavia, A., Robinson, G.G.N., Bradley, B., Ressler, K.J., Powers, A., 2016. Exposure to Childhood Abuse and Later Substance Use: Indirect Effects of Emotion Dysregulation and Exposure to Trauma. J. Trauma. Stress 29, 422–429. https://doi.org/10.1002/jts.22131

Petit, G., Luminet, O., Maurage, F., Tecco, J., Lechantre, S., Ferauge, M., Gross, J.J., de Timary, P., 2015. Emotion Regulation in Alcohol Dependence. Alcohol. Clin. Exp. Res. 39, 2471–2479. https://doi.org/10.1111/acer.12914

Preacher, K.J., MacCallum, R.C., 2003. Repairing Tom Swift’s Electric Factor Analysis Machine. Underst. Stat. 2, 13–43. https://doi.org/10.1207/S15328031US0201_02

Schoemann, A.M., Boulton, A.J., Short, S.D., 2017. Determining Power and Sample Size for Simple and Complex Mediation Models. Soc. Psychol. Personal. Sci. 8, 379–386. https://doi.org/10.1177/1948550617715068

Strickland, J.C., Johnson, M.W., 2020. Rejecting impulsivity as a psychological construct: A theoretical, empirical, and sociocultural argument. Psychol. Rev. https://doi.org/10.1037/rev0000263

Verbruggen, F., Aron, A.R., Band, G.P., Beste, C., Bissett, P.G., Brockett, A.T., Brown, J.W., Chamberlain, S.R., Chambers, C.D., Colonius, H., Colzato, L.S., Corneil, B.D., Coxon, J.P., Dupuis, A., Eagle, D.M., Garavan, H., Greenhouse, I., Heathcote, A., Huster, R.J., Jahfari, S., Kenemans, J.L., Leunissen, I., Li, C.-S.R., Logan, G.D., Matzke, D., Morein-Zamir, S., Murthy, A., Paré, M., Poldrack, R.A., Ridderinkhof, K.R., Robbins, T.W., Roesch, M., Rubia, K., Schachar, R.J., Schall, J.D., Stock, A.-K., Swann, N.C., Thakkar, K.N., van der Molen, M.W., Vermeylen, L., Vink, M., Wessel, J.R., Whelan, R., Zandbelt, B.B., Boehler, C.N., 2019. A consensus guide to capturing the ability to inhibit actions and impulsive behaviors in the stop-signal task. eLife 8, e46323. https://doi.org/10.7554/eLife.46323
